# The role of RST1 and RIPR proteins in plant RNA quality control systems

**DOI:** 10.1007/s11103-021-01145-9

**Published:** 2021-04-17

**Authors:** Mariann Auth, Tünde Nyikó, Andor Auber, Dániel Silhavy

**Affiliations:** 1 Biological Research Centre, Institute of Plant Biology, ELKH, Temesvári krt 62, 6726 Szeged, Hungary; 2 Agricultural Biotechnology Institute, Department of Genetics, NARIC, Gödöllő, Hungary

**Keywords:** Plant RNA quality control, Endonucleolytic cleavage, Degradation of 5′cleavage fragments, Minimum ORF, 3′-5′ exonuclease

## Abstract

To keep mRNA homeostasis, the RNA degradation, quality control and silencing systems should act in balance in plants. Degradation of normal mRNA starts with deadenylation, then deadenylated transcripts are degraded by the SKI-exosome 3′-5′ and/or XRN4 5′-3′ exonucleases. RNA quality control systems identify and decay different aberrant transcripts. RNA silencing degrades double-stranded transcripts and homologous mRNAs. It also targets aberrant and silencing prone transcripts. The SKI-exosome is essential for mRNA homeostasis, it functions in normal mRNA degradation and different RNA quality control systems, and in its absence silencing targets normal transcripts. It is highly conserved in eukaryotes, thus recent reports that the plant SKI-exosome is associated with RST1 and RIPR proteins and that, they are required for SKI-exosome–mediated decay of silencing prone transcripts were unexpected. To clarify whether RST1 and RIPR are essential for all SKI-exosome functions or only for the elimination of silencing prone transcripts, degradation of different reporter transcripts was studied in RST1 and RIPR inactivated *Nicotiana benthamiana* plants. As RST1 and RIPR, like the SKI-exosome, were essential for Non-stop and No-go decay quality control systems, and for RNA silencing- and minimum ORF-mediated decay, we propose that RST1 and RIPR are essential components of plant SKI-exosome supercomplex.

**Supplementary Information:**

The online version contains supplementary material available at 10.1007/s11103-021-01145-9.

## Key message

The RST1 and RIPR proteins are required for the degradation of aberrant transcripts lacking a stop codon and the 5′ cleavage fragments of no-go decay, RNA silencing and minimum ORF.

**Supplementary Information:**

The online version contains supplementary material available at 10.1007/s11103-021-01145-9.

## Introduction

Eukaryotic mRNA degradation starts with deadenylation. Dissociation of the poly(A) binding protein (PABP) from the deadenylated mRNA promotes decapping, and then the unprotected transcript is quickly degraded by XRN4 cytoplasmic 5′-3′ exonuclease (in yeast and animals XRN1) and/or by 3′-5′ exonucleases, mainly by the conserved SKI-exosome (see below) (Houseley and Tollervey 2009; Li et al. 2018; Tatosyan et al. 2020). In plants, both 5′-3′ and 3′-5′ exonucleases play an important role in the control of mRNA stability (Sieburth and Vincent 2018; Sorenson et al. 2018). In addition to the elimination of the normal transcripts, the XRN4 and SKI-exosome exonucleases also play an important role in cytoplasmic RNA quality control systems. These systems identify aberrant transcripts and induce their quick decay. The Nonsense-mediated decay (NMD), the Non-stop decay (NSD) and the No-go decay (NGD) are translation-coupled quality control systems (Karamyshev and Karamysheva 2018; Urquidi Camacho et al. 2020), while RNA silencing quality control system degrades both translated and non-translated transcripts (Lee et al. 2020). Plant NMD degrades a transcript if its 3′UTR is unusually long or contains an intron more than 50 nt downstream from the stop codon (Arciga-Reyes et al. 2006; Kertész et al. 2006; Schwartz et al. 2006; Gloggnitzer et al. 2014). NGD and NSD are related systems although they target different transcripts. NGD degrades faulty mRNAs harboring a translation elongation blocking element, while NSD eliminates transcripts lacking an in-frame stop codon (nonstop mRNAs). In plants, long A-stretches can block elongation and the stalled ribosomes activate NGD (Szádeczky-Kardoss et al. 2018b). It cleaves the transcript upstream of the A-stretch, and then the 3′ and 5′cleavage fragments are degraded by the XRN4 and the SKI-exosome, respectively. The Pelota and the HBS1 proteins, the paralogs of the eRF1 (eukaryotic Release Factor1) and eRF3 translation termination factors (Atkinson et al. 2008), are also required for the decay of the 5′ cleavage fragment (Szádeczky-Kardoss et al. 2018b). After NGD cleavage the next ribosome runs to the 3′ end of the 5′ cleavage product. The Pelota binds to the empty A-site of this ribosome and (with the help of HBS1) initiates ribosome splitting, thereby promoting the SKI-exosome mediated decay of the 5′ cleavage product. NSD might operate similarly, in the absence of the stop codon the ribosome runs into the poly(A) tail, which induces transcript cleavage upstream (Guydosh and Green 2017). The Pelota-HBS1 complex as well as the SKI-exosome are required for the decay of the NSD target transcripts in plants (Szádeczky-Kardoss et al. 2018a). The molecular basis of NGD induction has been studied in yeast and animals. It was shown that collision of two or more ribosomes leads to ribosome ubiquitination, which results in NGD-mediated transcript cleavage (Simms et al. 2017; Juszkiewicz et al. 2018; Ikeuchi et al. 2019; Navickas et al. 2020;). NGD activation is position-dependent in yeast, translation blocking sequences induce NGD-mediated cleavage only if they are present at least ~ 100 nt from the start codon, likely because a critical distance is essential for effective ribosome collisions (Simms et al. 2017). It is not known how NGD is activated in plants.

Although RNA silencing quality control system originally functioned against molecular parasites, it has evolved into a sophisticated gene regulatory system (Shabalina and Koonin 2008). Silencing is triggered by double-stranded RNA (dsRNA) and causes degradation or translational repression of the homologous mRNAs (Fire et al. 1998). Plant dsRNAs are processed by the DICER-LIKE proteins (DCLs) into 21–24 nt small RNAs (sRNAs) (Bernstein et al. 2001; Park et al. 2002; Reinhart et al. 2002). microRNAs (miRNAs) and small interfering RNAs (siRNAs) are the most important plant sRNAs (Fang and Qi 2015). sRNAs are loaded onto one of the AGO proteins forming the RNA-Induced Silencing Complex (RISC), and guide the RISC to the complementary nucleic acids for silencing. Plant RISCs mostly cleave target mRNAs within the coding region (Reinhart et al. 2002; Poulsen et al. 2013). The 5′ and 3′ cleavage products can be degraded by exonucleases or become substrates to RNA-dependent RNA polymerase (RDR)-mediated silencing amplification (also called transitivity) (Souret et al. 2004; Branscheid et al. 2015; Zhang et al. 2018). In *Arabidopsis*, RDR6 can convert RISC cleavage fragments into dsRNAs, from which DCLs process secondary siRNAs. These guide RISC to target transcripts, thereby amplifying the silencing response. Silencing amplification plays a critical role in antiviral responses (Mourrain et al. 2000; Yang and Li 2018). Aberrant transgenic transcripts also induce silencing amplification. In contrast, endogenous mRNAs and the cleavage products of miRNA-programmed RISC (miRISC) do not trigger amplification in wild-type plants (de Felippes and Waterhouse 2020; Tan et al. 2020). However, if any of the RNA degradation systems, the decapping-XRN4, the SKI-exosome or the NMD system is impaired (and especially if both the XRN4 and the SKI-exosome exonucleases are inactivated) detrimental secondary siRNAs (called rqc- or ct-siRNAs) are generated from silencing prone mRNAs and miRNA targets (Mourrain et al. 2000; Souret et al. 2004; Moreno et al. 2013; Branscheid et al. 2015; De Alba et al. 2015; Lam et al. 2015; Wu et al. 2020; Zhang et al. 2015; Zhao and Kunst 2016). Thus RNA silencing and the different RNA degradation systems should act in balance to ensure sRNA and mRNA homeostasis (Liu and Chen 2016; Sieburth and Vincent 2018; Tan et al. 2020; de Felippes and Waterhouse 2020).

The exosome is a conserved 3′-5′ exonuclease that is recruited to the target transcripts by adaptor proteins (Januszyk and Lima 2014; Schmidt et al. 2016). The SKI complex (consisting of the SKI2, SKI3 and SKI8 proteins) is the only known cytoplasmic exosome adaptor (Mitchell 2014). It is associated with the 80S ribosome and its key factor, the SKI2 helicase unwinds and threads the mRNA to the exosome channel for decay (Schmidt et al. 2016). In yeast and mammals, the SKI7 protein connects the SKI and exosome complexes (Kalisiak et al. 2017; Schmidt et al. 2016) and SKI7 is also bound to both complexes in plants (Brunkard and Baker 2018; Lange et al. 2019). Recently it was proposed that in plants two additional proteins, the conserved RST1 and the plant specific RIPR are also involved in a SKI-exosome degradation pathway. Immunoprecipitation-MS assays suggest that RST1 and RIPR form a complex and that they also interact with the SKI and exosome complexes and the SKI7 protein (Lange et al. 2014, 2019). Moreover, RST1 and RIPR mutants show similar phenotype to the SKI and exosome mutants in that aspect that rqc-siRNAs were generated and silencing amplification was induced at silencing prone transcripts in RST1, RIPR, SKI and exosome mutants (Chen et al. 2005; Lange et al. 2019; Li et al. 2019; Daszkowska-Golec 2020; Yang et al. 2020). Thus RST1 and RIPR cooperate with the SKI-exosome system to degrade silencing amplification prone mRNAs. It was hypothesized that that the RST1-RIPR complex is also involved in other SKI-exosome activities including NGD and NSD quality control systems (Lange et al. 2019).

We have recently identified the *cis* and certain *trans* factors of the plant NGD and NSD systems (Szádeczky-Kardoss et al. 2018a, b; Szádeczky-Kardoss et al. 2018a, b). As endonuclease cleaved transcripts are sensitive to RNA silencing mediated rqc-siRNA generation, we wanted to study the *cis* requirement of NGD-mediated cleavage and tried to experimentally test the hypothesis that the RST1 and RIPR proteins play a role in NGD and NSD quality controls. We show that in plants, like in yeast, elongation blocking sequences activates NGD-mediated cleavage in a position-dependent manner. We also demonstrate that RST1 and RIPR are required to eliminate NSD target transcripts and NGD generated 5′cleavage fragments. Moreover, we found that RST1 and RIPR are also involved in the elimination of 5′cleavage fragments generated by miRNA or viral siRNA programmed RISC (miRISC, vsiRISC) and in the degradation of minimum ORF induced 5′ cleavage fragments. As we found that RST1 and RIPR are involved in all tested cytoplasmic SKI-exosome activities, we propose that these proteins are essential for the function of the SKI-exosome supercomplex in plants.

## Materials and methods

### Plasmid constructs

The P-36A-G and P-72A-G NGD, the PHAnst NSD reporter constructs, the PPG viral siRNA sensor, the GFP-amiRGFP system, the Pel2 Pelota dominant-negative construct, the TRV-PDS, TRV-P-Pel, TRV-P-HBS1, TRV-P-SKI2, TRV-P-XRN4 VIGS vectors and the P14 silencing suppressor were previously described (Mérai et al. 2005; Kertész et al. 2006; Nyikó et al. 2009; Szádeczky-Kardoss et al. 2018a, b; Szádeczky-Kardoss et al. 2018a, b). The P-36A-G and P-72A-G are similar fusion reporter constructs, in which the 281 nt long segment from the PHA (phytohemagglutinin) gene and the full-length GFP is separated with 36A and 72A sequences, respectively. To generate TRV-P-RST1 and TRV-P-RIPR VIGS vectors, 487 and 413 nt long PCR fragments were amplified with Nb Rst1 VIGS EcoRI F / Nb Rst1 VIGS EcoRI R and Nb Ripr VIGS EcoRI F / Nb Ripr VIGS EcoRI R primer pairs from *Nicotiana benthamiana* cDNA, and then the EcoRI cleaved fragments were cloned into TRV-PDS vector. The conserved upstream ORF (uORF) and the minimum ORF reporter constructs were cloned into Bin61S or its derivatives (Silhavy et al. 2002). Cloning details and the list of used primers are described at Supplementary Data 1.

### Identification of putative orthologs of *N. benthamiana* RST1 and RIPR

To identify the putative *N. benthamiana* orthologs of RST1 and RIPR, BLAST search was conducted with the *Arabidopsis* protein sequences on *N. benthamiana* predicted cDNA databases (solgenomics.net). It predicted two homologss for both proteins, Niben101Scf01448g02001 and Niben101Scf02585g00004 for RST1 and Niben101Scf04173g00001 and Niben101Scf04792g00001 for RIPR. The *N. benthamiana* RST1 and RIPR homologs were aligned to the corresponding *Arabidopsis* proteins. The more similar RST1 and RIPR copies (Niben101Scf01448g02001, Niben101Scf04173g00001) showed 45,1 and 32,2% identity and 62,4 and 48,1% similarity to the corresponding *Arabidopsis* proteins (Suppl. Fig. 2). RST1 and RIPR VIGS fragments were designed to target both copies (96–100% identity in the VIGS region). The RST1 and RIPR qRT-PCR primers also measure the expression of both *N. benthamiana* RST1 and RIPR copies.

### Agroinfiltration and VIGS-agroinfiltration assays

*Nicotiana benthamiana* plants were grown in the greenhouse. After agroinfiltration or VIGS treatment (see below) the plants were kept in a growth chamber at 23 °C under 16/8 light/dark condition.

Agroinfiltration transient gene expression was described previously (Silhavy et al. 2002; Krenek et al. 2015). Briefly, each construct is transformed into an *Agrobacterium tumefaciens* strain, and then *N. benthamiana* leaves were agroinfiltrated with a mixture of bacterium cultures (each culture was diluted to OD 0.4 except the P14, which was diluted to 0.2). The P14 silencing suppressor of *Pothos Latent Virus* (*Aureusvirus*) was always included in the mixture. Agroinfiltration triggers strong silencing response that could override the effect of RNA quality control. To prevent the induction of agroinfiltration triggered silencing, the P14 viral silencing suppressor is always co-expressed in agroinfiltration and VIGS-agroinfiltration assays. P14 does not modify the NGD, NSD, NMD, miRISC or vsiRISC cleavage experiments (Szádeczky-Kardoss et al. 2018a). The P14 can also be used as a control to measure the expression of the co-agroinfiltrated reporter construct.

VIGS-agroinfiltration assay is a combination of Virus-induced gene silencing (VIGS) transient gene inactivation (Dommes et al. 2019) and agroinfiltration transient gene expression systems. It is an efficient tool to identify components of RNA quality control systems in *N. benthamiana* (Kerényi et al. 2008; Szádeczky-Kardoss et al. 2018a, b). The VIGS-agroinfiltration assay consists of two steps; (1) VIGS is used to silence the putative RNA quality control factor, and (2) agroinfiltration mediated expression of an RNA quality control reporter construct in the VIGS silenced leaves. To initiate VIGS, ~ 21 days old *N. benthamiana* plants were co-agroinfiltrated with a mixture of three *Agrobacterium* cultures. One expressed P14, the second *Tobacco Rattle Virus* (TRV) RNA1 and the third expressed TRV RNA2 containing segments from *N. benthamiana* PDS (phytoene desaturase) gene or from PDS and a sequence from the target gene. Systemic TRV infection triggers efficient RNA silencing antiviral response that silences the virus and the endogen gene, whose fragment was inserted into the TRV RNA2 vector. PDS is used to monitor silencing as PDS silencing leads to photobleaching (leaf whitening). 10–14 days after VIGS inoculation (d.p.i), when the upper leaves are whitened (showing that PDS silencing was effective and suggesting that the silencing of the gene of interest is also effective), leaves under the top white ones were agroinfiltrated with *Agrobacterium* cultures expressing the RNA quality control test construct and the P14 control. From each VIGS type three plants, at which photobleaching indicated that silencing was efficient, were agroinfiltrated.

Quantitative RT-PCR (qRT-PCR) assays confirmed that the VIGS selectively and efficiently reduced PDS, SKI2, RST1 and RIPR target mRNAs, although SKI2 silencing was obviously more efficient than the RST1 or RIPR VIGS (Suppl. Fig. 1).

### Pel2 assay

To inhibit Pelota-HBS1 complex, *Arabidopsis* Pel2, the dominant-negative paralog of Pelota was co-agroinfiltrated with P14 and the test construct (Szádeczky-Kardoss et al. 2018b).

### RNA gel blot and qRT-PCR assays

RNA gel blot assays were described (Silhavy et al. 2002). Briefly, one leaf of three *N. benthamiana* plants (wild-type or VIGS plants, in which silencing was efficient) were agroinfiltrated with each Agrobacterium mixture. At 3 d.p.i, the agroinfiltrated leaves were collected. Total RNAs isolated from these samples were separated on denaturing agarose gel, blotted and hybridized with radioactively labeled probes. The blots were scanned with Molecular Imager PharosFX™ System (Bio-Rad) and were quantified with the ImageLab 6.0.1 Software. RNA gel blots that were used for the quantification are presented as supplementary figures and one set of samples presented as main figure. When accumulation of the 5′ cleavage products was measured, 5′ probe that hybridizes to both the full-length mRNA and the 5′ cleavage fragment was used for hybridization. The ratio of the 5′ cleavage fragment and the corresponding full-length transcript was calculated for each lane. The mean values and the standard deviations of the 5′fragment/full-length transcript ratios were calculated from three samples (one leaf from the three plants that were agroinfiltrated with the same mixture). Finally, the negative control was taken as 1 and the test and positive control samples were calculated relative to the negative control. At the non-stop decay experiment (PHAnst reporter) only the expression of the full-length transcript was measured, therefore the blot was also hybridized with P14 probe and the P14 signal was used as a control for quantifications. The quantification was similar to the 5′cleavage/full-length transcript quantifications (see above) except that the ratio of the full-length transcript/P14 transcript was calculated for each lane. For qRT-PCR assays, cDNA was synthesized with RevertAid First Strand cDNA Synthesis kit (Thermo Fisher Scientific) from DNAse I treated total RNA samples. qRT-PCR assays were carried out with Fast Start Essential DNA Green Master Mix (Roche) in a Light Cycler 96 (Roche) Real-Time PCR machine. Ubiquitin (Niben101Scf01956g01003.1) was used as an internal control for qRT-PCR assays.

## Results

### The efficiency of long A-stretch induced NGD depends on the distance from the start codon

**Fig. 1:**
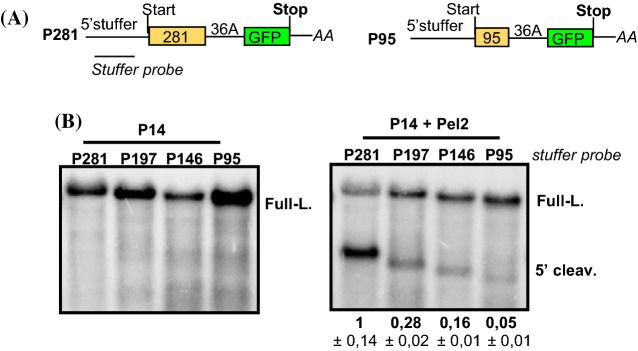
The position of the A-stretch is critical for NGD induction. **a** Non-proportional representation of two reporter transcripts used in the experiment. In the P281 NGD reporter mRNA a 36A stretch separates the 281 nt long PHA segment from the GFP. The 5′ UTR was extended by cloning a stuffer segment into it. P95 is a deletion derivative of P281, the PHA segment was shortened to 95 nt. P197 and P146 constructs (not shown) are identical except that the length of the PHA segment is different. Note that reporter transcripts and not reporter genes are shown. **b** NGD efficiency depends on the position of the NGD inducing A-stretch. Reporter genes were co-agroinfiltrated with P14 (left panel) or with P14 and Pelota2 (Pel2), a dominant-negative version of Pelota (right panel). Three plants were agroinfiltrated with each mixture (*n* = 3) and RNA was isolated from one leaf of each agroinfiltrated plant. RNA gel blots were hybridized with 5′ UTR probes (stuffer probe, shown in italics). Full-L. shows the full-length mRNAs, while 5′ cleav. indicates the NGD generated and Pel2 stabilized 5′cleavage fragments. One sample from each agroinfiltration is presented here. RNA gel blots, which present all RNA samples and were used for quantifications are shown as Suppl. Figure 3. To quantify the RNA gel blot, the ratio of the 5′ cleavage product and the full-length transcript (5′ cleav./Full-l.) were calculated for each lane from the Pel2 co-agroinfiltrated samples (5′ cleavage fragments cannot be detected in the absence of Pel2, thus that blot was not quantified). The mean values and the standard deviations were calculated for each agroinfiltration mixture from the three samples. Then the average of the P281 sample was taken as 1 and the mean values and standard deviation of other samples were normalized to it (shown under the panel)

Previously we have shown that a long A-stretch in the coding region induces No-go decay (NGD) mediated mRNA cleavage in plants. The efficiency of NGD activation depends on the length of the A-stretch, the longer the A-stretch, the more efficient the cleavage (Szádeczky-Kardoss et al. 2018b). Recent results suggest that in yeast the efficiency of NGD mediated cleavage also depends on the distance between the A-stretch and the start codon (Simms et al. 2017). To test whether plant NGD is also sensitive to the position of the A-stretch, we altered the position of the A-stretch and then assessed the intensity of NGD by studying the accumulation of the 5′ cleavage fragments. We used a previously described NGD fusion reporter construct (P-36A-G), in which a 281 nt long segment from the PHA (phytohemagglutinin) gene and the full-length GFP is separated with 36A sequence (Szádeczky-Kardoss et al. 2018b). To facilitate the detection of the 5′ fragments, we extended the 5′UTR by incorporating a 357 nt long stuffer region (Nyikó et al. 2009) that does not contain start codon (the final construct was called P281). To modify the position of the A-stretch relative to the start codon, the 281 nt PHA segment was shortened to 197, 146 and 95 nt (named: P197, P146, P95) (Fig. 1a). These constructs were co-agroinfiltrated into *N. benthamiana* leaves with only P14 or were co-agroinfiltrated with P14 and the dominant-negative version of Pelota (Pel2). Agroinfiltration induces strong RNA silencing response that could degrade the NGD reporter transcript. Expression of the P14 viral silencing suppressor prevents silencing induction. Pel2, which inhibits the Pelota-HBS1 complex, is co-agroinfiltrated to stabilize the 5′ cleavage fragment of NGD (Mérai et al. 2005; Szádeczky-Kardoss et al. 2018a, b). RNA gel blots were hybridized with 5′UTR probe to monitor the accumulation of the 5′ cleavage fragments. In the absence of Pel2 the 5′ NGD cleavage fragments were not detected (Fig. 1b left panel). By contrast, 5′ cleavage products accumulated to detectable levels from all four (P281, P197, P146 and P95) constructs when Pel2 was co-agroinfiltrated. As Pel2 inhibits the degradation of the 5′ cleavage fragment, the accumulation of 5′ product indicates the efficiency of NGD mediated cleavage. Relevantly, the efficiency of cleavage was very different, the 5′ NGD cleavage fragments were abundant at the P281 constructs, moderate and weak at the P197 and P146 constructs and were barely detectable at the P95 construct (Fig. 1b right panel and Suppl. Fig. 3). Thus we concluded that in plants, like in yeast, the NGD induction depends on the position of the activating element, the longer the distance from the start codon, the more efficient the NGD.

### RST1 and RIPR play a role in the degradation of 5′ fragments of NGD induced cleavage

**Fig. 2:**
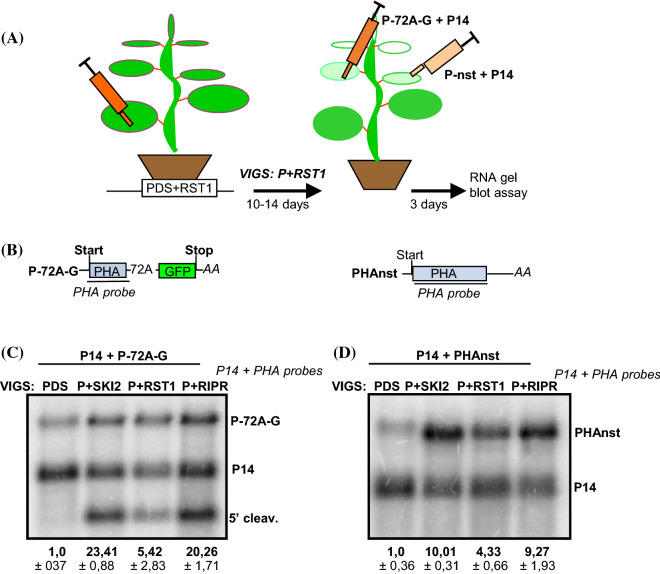
The RST1 and the RIPR are involved in the degradation of the 5′ cleavage fragments of NGD and in the elimination of NSD target mRNAs. **a** The VIGS-agroinfiltration system to study the role of RST1 putative RNA quality control factor in NSD and NGD (for details see the main text). **b** Non-proportional representation of the PHA-72A-GFP (P-72A-G) NGD and the PHAnonstop (PHAnst) NSD reporter transcripts. **c** The RST1 and the RIPR are required for the decay of the 5′ fragments generated by NGD. P-72A-G NGD reporter construct, in which the PHA and GFP segments are separated with 72A sequence, was co-agroinfiltrated with P14 into a leaf of three PDS, PDS + SKI2 (P + SKI2), PDS + RST1 (P + RST1) and PDS + RIPR (P + RIPR) VIGS plants. RNA samples were extracted from the three agroinfiltrated leaves from each VIGS type (*n* = 3). RNA gel blots were hybridized with P14 and PHA probes (probes are in italics). P-72A-G indicates the full-length reporter mRNAs, while 5′ cleav. marks the 5′ NGD cleavage fragments. RNA gel blots, which present all RNA samples and were used for quantifications are shown as Suppl. Figure 4B. One sample from each agroinfiltration is presented here. To quantify the gel blot, the ratio of the cleavage product and the full-length transcript (5′ cleav./P-72A-G) were calculated for each lane. The mean values and standard deviations were calculated for each VIGS type. Then the average of the PDS samples was taken as 1 and the mean values and standard deviations of other lines were normalized to it. P14 is visualized but it was not used for the quantification. Note that 5′ cleavage fragments are overaccumulated in P + SKI2, P + RST1 and P + RIPR plants relative to the PDS control. **d** The RST1 and the RIPR are required for the elimination of NSD target transcript. PHAnonstop (PHAnst) NSD reporter construct was co-agroinfiltrated with P14 into a leaf of three PDS, P + SKI2, P + RST1 and P + RIPR VIGS plants. RNA gel blots, which present all RNA samples and were used for quantifications are shown as Suppl. Figure 4C. P14 was used for quantification. To quantify the RNA gel blot, the ratio of the nonstop reporter and the P14 control mRNA (PHAnst/P14) was calculated for each lane, then the mean values and the standard deviations were calculated for each VIGS type. The average of the PDS samples was taken as 1 and the mean values and standard deviations of other VIGS lines were normalized to it

The RST1 and RIPR proteins interact with the SKI-exosome and are involved in the degradation of RNA silencing sensitive mRNAs and miRNA targets (Lange et al. 2019; T. Li et al. 2019). It was hypothesized that these proteins also play role in other cytoplasmic SKI-exosome activities (Lange et al. 2019). Previously we and others have shown that SKI-exosome is required for the degradation of Non-stop decay (NSD) target transcripts and for the elimination of the 5′ endonucleolytic cleavage fragments generated by NGD, miRNA or viral siRNA programmed RISC (miRISC, vsiRISC) (Branscheid et al. 2015; Szádeczky-Kardoss et al. 2018a, b; Szádeczky-Kardoss et al. 2018a, b). To experimentally test whether RST1 and RIPR are also involved in these decays, VIGS-agroinfiltration assays were conducted. The putative *N. benthamiana* RST1 and RIPR genes were identified, and then Virus-induced gene silencing (VIGS) transient gene inactivation system was used to generate RST1 and RIPR silenced *N. benthamiana* plants. The NSD, NGD or RISC reporter constructs were agroinfiltrated into the silenced leaves and the accumulation of reporter transcripts was studied by RNA gel blot assays (Fig. 2a). If RST1 and RIPR are required for the degradation of NSD target transcripts or for the elimination of 5′ cleavage fragments of NGD or RISC, the corresponding reporter transcripts will overaccumulate in these silenced plants.

First we tested if RST1 and RIPR are involved in NGD. To address this issue, four different VIGS plants were generated, and then a NGD reporter construct was expressed in these plants. In the negative control *N. benthamiana* VIGS plant only the PDS (phytoene desaturase) gene was silenced, while in the positive control plant the PDS and the SKI2 genes were co-silenced. We also generated two test VIGS plants, in which the PDS and the RST1 or the PDS and the RIPR genes were co-silenced (referred to as PDS, P + SKI2, P + RST1 and P + RIPR plants, respectively). As silencing of PDS leads to photobleaching, leaf whitening could be used to monitor the efficiency of VIGS mediated gene inactivation. When whitening of the top leaves showed that VIGS is efficient (~ 10–14 days after VIGS induction) a NGD reporter construct (P-72A-G), in which a 72nt long A-stretch separates the PHA and GFP sequences (Fig. 2a) was co-agroinfiltrated into the silenced *N. benthamiana* leaves with P14 silencing suppressor (P14 inhibits silencing induction but does not effect on VIGS efficiency). RNA gel blot assays were carried out to monitor the accumulation of 5′ NGD cleavage products. In line with our previous results we found that the 5′ NGD cleavage fragments accumulated to high levels in the P + SKI2 (Fig. 2c) positive control plants but not in the PDS silenced negative control plant. Relevantly, the 5′ NGD cleavage fragments also accumulated to high levels in the P + RST1 and P + RIPR test plants (Fig. 2c and Suppl. Fig. IVB). Thus, RST1 and RIPR, like the SKI2, are not required for the endonucleolytic cleavage step of NGD but they are involved in the elimination of NGD generated 5′ cleavage fragments.

### RST1 and RIPR are required for the elimination of NSD target transcripts

Nonstop aberrant transcripts, which lack an in-frame stop codon but contain poly(A) tail, are targeted by the NSD system. The SKI-exosome complex is required for the elimination of nonstop transcripts in plants (Szádeczky-Kardoss et al. 2018a). To clarify the role of RST1 and RIPR in plant NSD, a nonstop reporter construct (PHAnst) was co-agroinfiltrated with P14 into the leaves of P + RST1 and P + RIPR test and PDS and P + SKI2 control VIGS plants (Fig. 2a). As expected the NSD reporter mRNA was easily detectable in the leaves of P + SKI2 positive control plant. The reporter transcript also accumulated to high levels in both P + RST1 and P + RIPR silenced leaves relative to the PDS silenced control. These data indicate that RST1 and RIPR, like SKI2, play an important role in plant NSD (Fig. 2d and Suppl. Fig. IVC).

### RST1 and RIPR are involved in the degradation of 5′ cleavage fragments of miRNA and viral siRNA programmed RISC

**Fig. 3:**
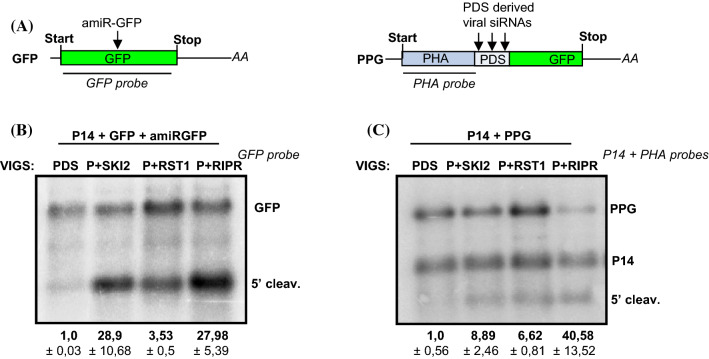
The RST1 and the RIPR play a role in the elimination of the 5′ fragments of miRISC and vsiRISC mediated cleavage. **a** Non-proportional representation of the reporter transcripts used in these experiments. **b, c** The RST1 and the RIPR are involved in the degradation of the 5′ fragments of miRISC or vsiRISC. GFP miRISC reporter was co-agroinfiltrated with amiRGFP and P14, while PHA-PDS-GFP (PPG) vsiRISC reporter was co-agroinfiltrated with P14 into PDS, PDS + SKI2 (P + SKI2), PDS + RST1 (P + RST1) and PDS + RIPR (P + RIPR) VIGS plants (*n* = 3). GFP and PPG indicate the full-length reporter mRNAs, while 5′ cleav. shows the 5′ cleavage fragments. RNA gel blots, which present all RNA samples and were used for quantifications are shown as Suppl. Figure 5B and C. RNA gel blots were quantified as described at Fig. 2c. Note that while P14 is shown at Fig. 3D, it was not used for quantification

Next we studied the role of RST1 and RIPR in the elimination of miRISC generated 5′ cleavage fragments. P14 was co-agroinfiltrated with GFP and a GFP targeting artificial miRNA (amiRGFP) into the leaves of PDS, P + SKI2, P + RST1 and P + RIPR VIGS plants (Fig. 3a). The miRNA that is processed from the amiRGFP transcript can incorporate into RISC, and then this miRISC cuts the GFP transcript in the coding region. In line with our previous results we found that the 5′ miRISC cleavage product was barely detectable in the PDS silenced negative control VIGS plant, while it accumulated to high levels in the P + SKI2 silenced positive control plant. We also found that the 5′ miRISC cleavage fragments were easily detectable in the P + RST1 and P + RIPR silenced plants (Fig. 3b and Suppl. Figure 5B).

To test whether the RST1 and RIPR are also involved in the degradation of 5′ endonucleolytic cleavage fragments generated by viral siRNA programmed RISC (vsiRISC), the PHA-PDS-GFP (PPG) vsiRISC sensor construct was expressed in the leaves of PDS, P + SKI2, P + RST1 and P + RIPR VIGS plants (Fig. 3a). PPG is a fusion construct, in which a PDS sequence is inserted between the PHA and GFP sequences. As PDS was silenced in all VIGS lines, viral PDS derived siRNA programmed RISCs (vsiRISC) are present in the leaves of these plants. Thus the PPG transcripts are cleaved by vsiRISC in the PDS region in all VIGS plants. RNA gel blot assays demonstrated that 5′ cleavage fragments accumulated in the P + RST1 and P + RIPR silenced test as well as in the P + SKI2 silenced positive control VIGS plants but not in the PDS silenced negative control VIGS plants (Fig. 3c and Suppl. Figure 5C). These data suggest that the RST1 and RIPR proteins are involved in the elimination of 5′ cleavage fragments generated by either miRISC or vsiRISC.

### SKI2, RST1 and RIPR, but not the Pelota-HBS1, are involved in the degradation of 5′ fragment of Minimum uORF activated cleavage

**Fig. 4:**
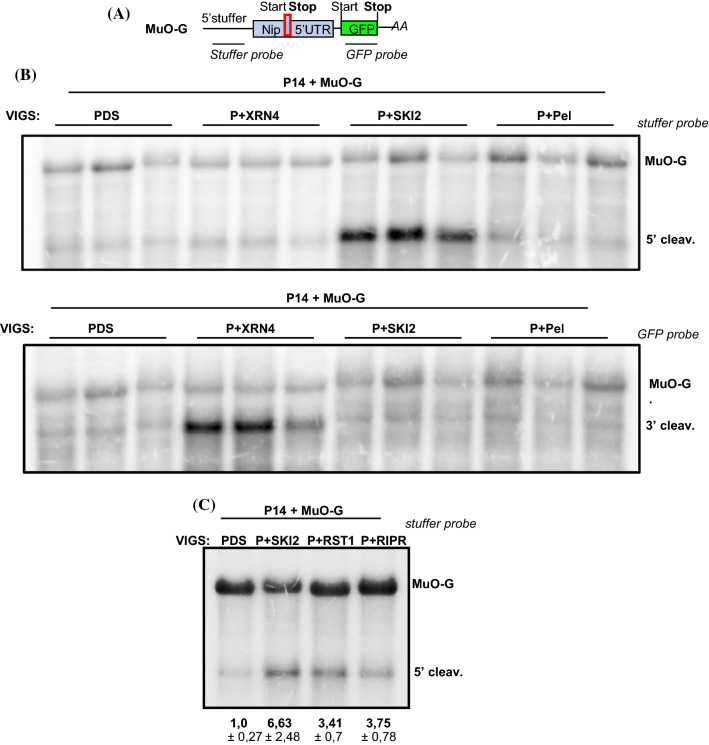
The SKI2, the RST1 and the RIPR are required for the elimination of the 5′ fragments of Minimum ORF induced cleavage. **a** Non-proportional representation of the Minimum ORF reporter mRNA (MuO-G). Minimum ORF (red box) containing 5′ UTR of the *Arabidopsis* NIP5 was cloned upstream of GFP. **b** The SKI2 and the XRN4 are required for the elimination of the 5′ and 3′ fragments of Minimum ORF induced cleavage. MuO-G was co-agroinfiltrated with P14 into a leaf of three PDS, PDS + XRN4 (P + XRN4), PDS + SKI2 (P + SKI2) and PDS + Pelota (P + Pel) VIGS plants (*n* = 3). Stuffer and GFP probes (italics) were used to visualize the full-length reporter mRNAs (MuO-G) and the 5′ and 3′ cleavage (5′ cleav. and 3′cleav.) fragments. Note that the same RNA gel blots with Ethidium-Bromide stained loading controls are shown as Suppl. Figure 7. **c** The RST1 and the RIPR are required for the elimination of the 5′ fragments of Minimum ORF induced cleavage. MuO-G was co-agroinfiltrated with P14 into PDS, P + SKI2, PDS + RST1 (P + RST1) and PDS + RIPR (P + RIPR) VIGS plants (*n* = 3). RNA gel blots, which present all RNA samples and were used for quantifications are shown as Suppl. Figure 9B. The RNA gel blots were quantified as described at Fig. 2c. Note that the 5′cleavage fragment of MuO-G accumulates to low but detectable levels even in the PDS VIGS plants

We also wanted to study how the cleavage fragments are degraded when the endonucleolytic cleavage occurs in the 5′UTR. It was reported that AUG-stop minimum uORF (MinuORF), which is present in the 5′ UTR of few genes including the key boron metabolism factor NIP5, trigger in boron concentration dependent manner a cleavage upstream of the MinuORF (Tanaka et al. 2016). Moreover, using in vitro translation assays it was shown that conserved plant uORFs (CuORF) cause ribosome stalling at the elongation or termination step of uORF translation (Tanaka et al. 2016; Hayashi et al. 2017). To study whether ribosome stalling at the CuORFs leads to endonucleolytic cleavage and to reveal how the cleavage fragments are eliminated, four CuORF containing 5′UTRs that triggered ribosome stalling in in vitro translation experiments and the MinuORF harboring 5′UTR of the *Arabidopsis* NIP5 were cloned upstream of the GFP reporter gene (Fig. 4a and Suppl. Figure 6a). These constructs were agroinfiltrated into the leaves of PDS silenced negative control and into the leaves of PDS and XRN4 (P + XRN4) co-silenced test VIGS plants. As XRN4 is the only known cytoplasmic 5′-3′ exonuclease, if these 5′UTRs lead to cleavage, their 3′ cleavage products can be detected in the P + XRN4 plants. We failed to detect 3′ cleavage fragments when the conserved uORF containing reporters were expressed in the P + XRN4 silenced plants (Suppl. Figure 6). These results suggest that under our *in planta* conditions, these CuORF containing 5′UTRs do not trigger endonucleolytic cleavage. By contrast, the 3′ cleavage fragment of the NIP5 MinuORF containing GFP reporter transcript (MuO-G for Minimum upstreamORF GFP) was abundant in the P + XRN4 but not in the PDS silenced plants (Suppl. Figure 6 and Fig. 4b bottom panel). These data confirm previous results that MuO-G mRNA is efficiently cleaved in agroinfiltrated *N. benthamiana* leaves and that the 3′ fragment is eliminated by XRN4 (Tanaka et al. 2016). We also found that the 5′ cleavage product of the MuO-G reporter mRNA accumulated to high levels in the P + SKI2 and to low levels in the P + XRN4 and PDS VIGS plants (Fig. 4b upper panel and Suppl. Figure 7). When the start codon of the minimum ORF was eliminated, the mRNA was not cleaved confirming that the minimum ORF induces the cleavage of the MuO-G mRNA (Suppl. Figure 7 right panels). Taken together, our data suggest that the 3′ and 5′ cleavage fragments of MuO-G mRNA are degraded by the XRN4 and SKI-exosome, respectively.

The Pelota-HBS1 complex cooperates with the SKI-exosome to eliminate the nonstop transcripts and the 5′ cleavage fragments of the RNA silencing and NGD systems (Szádeczky-Kardoss et al. 2018a, b). To test whether the Pelota-HBS1 complex also collaborates with the SKI-exosome complex to decay the 5′ cleavage fragments of MinuORF, the MuO-G reporter was expressed in VIGS plants in which the PDS + Pelota or the PDS + HBS1 genes were co-silenced (P + Pel and P + HBS1). Relevantly, the 5′ cleavage fragments were not overaccumulated (relative to the PDS control) in these silenced plants (Fig. 4b upper panel, Suppl. Figures 7 and 8). Moreover, the 5′ cleavage fragment did not overaccumulate when the Pelota-HBS1 complex was inactivated by overexpressing Pel2 (Suppl. Figure 8), a dominant-negative paralog of Pelota (Szádeczky-Kardoss et al. 2018b). As the 5′ cleavage fragment of P281 NGD reporter (Fig. 1) accumulated to high levels when Pel2 was co-expressed as well as in the P + HBS1 VIGS plants, it is likely that the Pelota-HBS1 inactivation was physiologically relevant (Suppl. Fig. 8).

Next we asked if the RST1 and RIPR proteins are required for the degradation of the 5′ fragments generated by MinuORF induced cleavage. We found that the level of the 5′ cleavage products of the MinuORF reporter mRNA was higher in the P + RST1 and P + RIPR test as well as in the P + SKI2 positive control VIGS plants than in the PDS silenced negative control plants (Fig. 4c and Suppl. Figure 9). Thus we concluded that the RST1 and RIPR proteins and the SKI-exosome complex are required for the decay of the 5′ fragments of the MinuORF induced cleavage, while the Pelota-HBS1 complex is likely not involved in the elimination of the 5′ cleavage products of the MinuORF.

Taken together, using transient reporter gene assays we demonstrated that in *N. benthamiana* plants, the RST1 and RIPR genes, like the SKI-exosome supercomplex, are involved in the decay of 5′ cleavage fragments of NGD, miRISC, vsiRISC or MinuORF induced cleavage, and for the elimination of NSD target nonstop transcripts.

## Discussion

### RST1 and RIPR play an important role in cytoplasmic SKI-exosome activities

Previously we have demonstrated that the SKI-exosome complex plays a critical role in different cytoplasmic RNA quality control systems in plants. We have found that SKI2 is required for the degradation of NSD target transcripts and for the elimination of 5′ cleavage fragments generated by NGD, miRISC or vsiRISC (Szádeczky-Kardoss et al. 2018a, b). It was demonstrated that the RST1 and RIPR proteins are bound to the SKI-exosome complexes and that in the RST1 and RIPR mutants, like in the SKI and exosome mutants, rqc-siRNAs are generated from silencing prone transcripts (Lange et al. 2019; Li et al. 2019; Yang et al. 2020). To distinguish between the possibilities that (1) RST1 and RIPR are only involved in the degradation of these silencing prone transcripts or that (2) they play a more general role in 3′-5′ degradation and are required for other SKI-exosome activities, RNA quality control reporter constructs were expressed in RST1 and RIPR silenced *N. benthamiana* plants. We found that the RST1 and RIPR proteins play a critical role in all previously identified SKI-exosome activities, these proteins were involved in the degradation of NSD target transcripts and in the elimination of 5′ cleavage fragments generated by NGD, miRISC or vsiRISC. Moreover, we found that SKI2, RST1 and RIPR are required for the efficient decay of 5′ fragments generated by MinuORF induced cleavage (Figs. 2, 3, 4). These results are consistent with the model that the RST1 and RIPR proteins, in addition to the SKI and exosome complexes, are critical components of the 3′-5′ degradation supercomplex (Lange et al. 2019).

In mammals, the SKI-exosome complex mainly involved in RNA surveillance, while XRN1 plays a predominant role in the degradation of normal transcripts (Tuck et al. 2020). Relevantly, we tested activities in which the SKI-exosome and the RST1-RIPR played surveillance function to eliminate the 5′ product generated by an endonucleolytic cleavage. However, based on indirect evidence it was proposed that in plants 3′-5′ exonucleases including SKI-exosome system also plays an important role in the degradation of normal mRNAs (Sorenson et al. 2018). Further studies are required to clarify the role of SKI-exosome and the RST1 and RIPR proteins in the bulk mRNA decay.

### The Pelota-HBS1 complex is not required for MinuORF induced mRNA decay

The Pelota-HBS1 proteins are also required for NSD and for the degradation of 5′ cleavage fragments generated by NGD, miRISC or vsiRISC (Szádeczky-Kardoss et al. 2018a, b), but it appears that they are not required for the elimination of 5′ fragments of MinuORF induced cleavage (Fig. 4b, Suppl. Figures 7 and 8). The proposed main role of Pelota-HBS1 complex is to remove the 80S ribosome from the 3′end of the 5′cleavage products of NGD, NSD or RISC, thereby allowing the SKI-exosome system to degrade these fragments. Indeed they are essential for target degradation when miRISC mediated cleavage occurs in the coding region but not when the cleavage happens in the 3′UTR (Szádeczky-Kardoss et al. 2018a, b; Szádeczky-Kardoss et al. 2018a, b). The MinuORF induced cleavage occurs 13 nt upstream of the P-site in the 5′ UTR (Tanaka et al. 2016). Thus it is likely that a scanning ribosome runs to 3′ end of the 5′ fragment at the MinuORF induced cleavage. We propose that the Pelota-HBS1 complex is not required to remove the scanning ribosome from the 3′ end of the 5′ MinuORF cleavage fragments but it is essential to disassemble the 80S ribosome from the 3′ end of the 5′ cleavage products generated by NGD, NSD and RISC. However, as silencing is never complete, we formally cannot exclude that the reduced amount of Pelota-HBS1 complex in the VIGS plants is sufficient to remove the scanning ribosome but not the 80S ribosome from the 3′ end of the different 5′ cleavage fragments.

### The putative role of RST1 and RIPR

Co-immunoprecipitation experiments revealed that RST1 is strongly associated with RIPR, SKI7 and the exosome, while RIPR mainly purifies with the SKI complex in addition to RST1 and SKI7 (Lange et al. 2019). It was proposed that the RST1 and RIPR form a complex that links the SKI and exosome complexes. Our result that both RST1 and RIPR were required for all tested SKI-exosome activities is consistent with this model. In yeast and mammals, SKI7 links the SKI and the exosome complexes and it is essential for the SKI-exosome activities (Schmidt et al. 2016; Kalisiak et al. 2017). SKI7 can also be co-immunoprecipitated with the SKI complex, exosome, RST1 and RIPR in plants (Lange et al. 2019). This observation is apparently in line with the assumption that in plants SKI7 also links the SKI and exosome complexes. However, it is likely that in plants, unlike in yeast and mammals, SKI7 is not essential for SKI-exosome mediated decay. Bioinformatical analyses suggest that in *Arabidopsis* SKI and HBS1 are generated from a single transcript by alternative splicing, while in *N. benthamiana* they are encoded by paralog genes (Brunkard and Baker 2018; Marshall et al. 2018). HBS1 VIGS in *N. benthamiana* led to impaired NSD and inefficient degradation of 5′ cleavage fragments generated by NGD or RISC (Suppl. Figure 8 and (Szádeczky-Kardoss et al. 2018a, b). Relevantly, the VIGS fragment that is used to inactivate HBS1 theoretically targets both the HBS1 and SKI7 coding *N. benthamiana* transcripts. However complementation assays showed that only HBS1 was required for the RNA quality control functions. Introduction of a construct that expresses the *Arabidopsis* HBS1 (but not the SKI7) mRNA isoform into the leaves of the HBS1 VIGS plants restored NSD activity and the decay of 5′ endonucleolytic cleavage fragments (Szádeczky-Kardoss et al. 2018a, b). Moreover, the HBS1 VIGS plants could be also complemented with overexpressing Pelota, the critical component of the Pelota-HBS1 complex. These results suggest that SKI7 is not essential for SKI-exosome mediated surveillance and that HBS1 plays a role in these activities as a component of the Pelota-HBS1 complex. It is tempting to speculate that SKI7 is not essential for SKI-exosome activities because in plants RST1 and RIPR can link the SKI and exosome complexes even in the absence of SKI7. RIPR is present only in flowering plants, while RST1 is a highly conserved protein that can be identified in metazoan (but not in fungi). The human RST1 (called FOCAD) forms a complex with AVEN and this complex binds to ribosomes and is transiently associated with the SKI complex (Tuck et al. 2020). However, the function of the FOCAD-AVEN and the RST1-RIPR complexes are likely different. RIPR and AVEN are non-related proteins and their knockout led to very different results. While RIPR inactivation inhibited SKI-exosome mediated surveillance in plants, AVEN knockout leads to increased SKI2V2L (the mammalian SKI2 ortholog) mRNA binding and intensified 3′-5′ decay. It is proposed that AVEN-FOCAD complex prevents ribosome stalling by melting translation blocking structures (Tuck et al. 2020).

We speculate that in the stem eukaryotes (the last common ancestor of extant eukaryotes) SKI7 connected the SKI and exosome complexes and the RST1/FOCAD was already associated with the SKI and/or the exosome complex. In different eukaryotic lineages the function of RST1/FOCAD diverged depending on the recruited partner. In metazoan branch, the RNA remodeler AVEN has become the partner of FOCAD and they act as anti-stalling factor. In the plant lineage, RST1 become associated with RIPR and they promote RNA surveillance activity of the SKI-exosome system by linking these complexes.

### NGD is induced in a position-dependent manner in plants

Previously we have shown that an A-stretch in the coding region triggers NGD mediated transcript cleavage in plants and that, the efficiency of cleavage depends on the length of the A-stretch (Szádeczky-Kardoss et al. 2018a, b). Here we demonstrated that the efficiency of NGD cleavage also depends on the position of the A-stretch (Fig. 1). The 5′ NGD cleavage fragments were barely detectable when the A-stretch (36A) was located 95 nt from the start codon but were abundant if it was present 197 or 281 nt from the start codon. Moreover, the NGD induction appears to be gradual in plants, the longer the distance from the start codon, the more efficient the NGD activation. NGD is also activated in a position-dependent manner in yeast, translation blocking sequences induce NGD-mediated cleavage only if they are present at least ~ 100 nt from the start codon (Simms et al. 2017). In yeast and mammals, collision of at least two or three translating ribosomes is required for NGD activation. Collision leads to ribosome ubiquitination, which results in NGD-mediated transcript cleavage upstream of the blocking sequence (Simms et al. 2017; Juszkiewicz et al. 2018; Ikeuchi et al. 2019; Navickas et al. 2020). These findings that ribosomes should collide to trigger NGD-mediated cleavage can explain the position dependency of NGD activation in yeast (Simms et al. 2017; Navickas et al. 2020). We speculate that ribosome collision is also a prerequisite for NGD activation in plants. We hypothesize that although collision of two or three ribosomes (which can occur if the A-stretch is located ~ 100 nt from the start codon) might be enough for weak NGD activation, collision of more ribosomes induces plant NGD more efficiently. It might explain the observation that efficacy of NGD cleavage is gradually increases with the distance from the start codon. It is still debated how and where the NGD cleavage occurs. It is proposed that ribosome ubiquitination leads to the recruitment of a specific endonuclease (CUE2 in yeast and NONU1 in worm) that cleaves at the colliding ribosome (D’Orazio et al. 2019; Glover et al. 2020). Alternatively, the cleavage occurs more upstream presumably by other nucleases (Navickas et al. 2020). It would be interesting to test whether weak and strong NGD induction leads to transcript cleavage at the same position in plants.

## Supplementary Information

Below is the link to the electronic supplementary material.

Supplementary file1 (PDF 1155 KB)
