# Supplementary material for: The role of RST1 and RIPR proteins in plant RNA quality control systems
Source: Plant Mol Biol. 2021 Apr 17;106(3):271–84. doi: 10.1007/s11103-021-01145-9 (PMC8116306; doi:10.1007/s11103-021-01145-9)

SUPPLEMENTARY DATA S1

I, Details of cloning

II, List of primers

III, Supplementary Figure Legends

IV, Supplementary figures

I, Details of cloning:

To create 780-G, HB1-G, SAUR-G and Hsfb1-G conserved uORF reporter constructs, the conserved uORF containing 5’UTR regions of AT1G70780, AT3G01470, At5G53590A and AT4G36990 genes were amplified from *Arabidopsis thaliana* genomic DNA with the corresponding (At780 5’UTR Kpn1 F / At780 5’UTR Kpn1 R, AtHB1 5’UTR Kpn1 F / AtHB1 5’UTR Kpn1 R, At SAUR30 5’UTR Kpn1 F / AtSAUR30 5’UTR Kpn1 R and AtHsfB1 5’UTR Kpn1 F / AtHsfB1 5’UTR Kpn1 R) primer pairs, and then the fragments were cloned with KpnI into the Bin61S-GFP vector upstream of the GFP. To create the NIP5-MinuORF-GFP reporter construct, 306 nt long region of the 5’UTR of *Arabidopsis* NIP5 gene (AT4G10380) was amplified with AtNIP5 5’UTR Kpn1 F / AtNIP5 5’UTR Kpn1 R primers and this fragment was incorporated into KpnI digested Bin61S-GFP. This 306 nt segment is sufficient for the MinuORF induced cleavage. To detect the 5’ cleavage products more easily, an 378 nt long stuffer sequence that did not contain AUG was amplified from the previously described 15-U2-noORF plasmid with NoATG stuffer SacI For /NoATG stuffer SacI Rev primer pair, and then it was cloned upstream of the 5’UTRs of the 780-G, HB1-G, SAUR-G and Hsfb1-G conserved uORF reporters constructs and the NIP5-MinuORF-G reporter (s780-G, sHB1-G, sSAUR-G, sHsfb1-G, Mu-O-G).

The NIP5 5’UTR region contains two uORFs, uORF1 and uORF2. uORF2 is sufficient to trigger Minimum ORF induced cleavage, thus it was used in this work (uORF2 is referred throughout the manuscript as minimum ORF, MinuORF). To prove that the MinuORF leads to cleavage, PCR mutagenesis was used to eliminate AUG of the MinuORF (No-uO-G). To generate No-uO-G plasmid, NoATG stuffer SacI For / AtNIP5 uORF nostart R and the AtNIP5 uORF nostart F / AtNIP5 5’UTR Kpn1 R primer pairs were used to amplify the modified PCR fragment from the Mu-O-G plasmid, then it was cleaved with SacI and KpnI

and cloned into the correspondingly cleaved Mu-O-G vector to replace the complete 5' UTR.

To test the position dependency of NGD induction, PCR fragments were amplified from P-36A-G plasmid with PHA100nt KpnI F, PHA150nt KpnI F, PHA200nt KpnI F or PHA KpnI F forward primers and with the GFP start 15 nt R reverse primer. The fragments were cloned with Acc65I into Mu-O-G vector between the stuffer and the GFP sequences.

II, List of primers

Primers used for cloning:

**At780 5'UTR Kpn1 F**	ATACAGGTACCTCTCTCTTAGCTCACCAACAATCAC
**At780 5'UTR Kpn1 R**	ACATAGGTACCCTTCGAAATCGAGAGAACAAAAAAC
**AtHB1 5'UTR Kpn1 F**	ATACAGGTACCGAGACGCTCACATTGCAAAAAC
**AtHB1 5'UTR Kpn1 R**	ACATAGGTACCTTTTGATCAACAGAAATAAAACAACAACTAC
**At SAUR30 5'UTR Kpn1 F**	ATACAGGTACCCCTAATTCCGAAACCACCTCCTC
**AtSAUR30 5'UTR Kpn1 R**	ACATAGGTACCTAAGAAGAAGGAATCTTTTTCACCG
**AtHsfB1 5'UTR Kpn1 F**	ATACAGGTACCTCTAGAAACAGCATCCGTTTTTATAAT
**AtHsfB1 5'UTR Kpn1 R**	ACATAGGTACCCTCCGGCGAACTTTTTTTATTTT
**AtNIP5 5'UTR Kpn1 F**	ACATAGGTACCATTTAAGTCCTAGCTCCATTTTCG
**AtNIP5 5'UTR Kpn1 R**	ACTATGGTACCTTCCAACGTTTTTTTTTTTGGTTTTTTT
**NoATG stuffer SacI For**	ACTATGAGCTCAGTAGTTAAAACAAGAACTGAAG
**NoATG stuffer SacI Rev**	TACATGAGCTCAGGTACCCAACTCTCTACCCA
**AtNIP5uORFnostartR**	GGAAATAAATTGATAGAGACGAAATTTACAGGATTTG
**AtNIP5 uORF nostart F**	CAAATCCTGTAAATTTCGTCTCTATCAATTTATTTCC
**PHA100nt KpnI F**	CATGGTACCATGGTGAGCTCTCTGGGCC
**PHA150nt KpnI F**	CATGGTACCATGGCCAAAGGCCAGTTACGACT
**PHA200nt KpnI F**	CATGGTACCATGGAAAGGTTCAACGAAACCAAC
**PHA KpnI F**	CATGGTACCATGGCTTCCTCCAACTTACTC
**GFP start 15 nt R**	GGGACAACTCCAGTGAAAAGTTC
**Nb Rst1 VIGS EcoRI F**	ATACGAATTCTGGACAAAGCTCCTAAAGCTGG
**Nb Rst1 VIGS EcoRI R**	ATACGAATTCAGATCAACTGGGATAAGGTCCTG
**Nb Ripr VIGS EcoRI F**	ATACGAATTCCGAAGCTCCCTTCGAATTGGG
**Nb Ripr VIGS EcoRI R**	TACAGAATTCGACGCCTCGATGCATGGTTC

Primers used for qRT-PCR

**Ubiquitin q F**	GCCGACTACAACATCCAGAAGG
**Ubiquitin q R**	TGCAACACAGCGAGCTTAACC
**Nb PDS q F**	TCTTGGCCGGAGCCAGAAGATG
**Nb PDS q R**	ATTCAGCGTGGCCGCCTTCA
**Nb RST1 q F**	TCAAGGAAGGCAGGAGCAGC
**Nb RST1 q R**	CCTCCGCATGAAGGGCTTCC
**Nb RIPR q F**	CATTGGCAAAGTCGAAGAGAGCTC
**Nb RIPR q R**	AGGTTTTTCCTTAACTTGCTTGCCA
**Nb Ski2 q F**	ACCAGAAGAGGCAGTGGCTA
**Nb Ski2 q R**	TTGGGCATACTCCTGTGGAT

III, Supplementary Figure Legends

Supplementary figure 1: The target mRNA levels are efficiently reduced in the VIGS plants. PDS transcript level was measured by qRT-PCR from the leaves of non-silenced (WT), the PDS-silenced (PDS), and the PDS+SKI2 (P+SKI2), PDS+RST1 (P+RST1) and PDS+RIPR (P+RIPR) VIGS co-silenced plants. The SKI2, RST1 and RIPR mRNA levels were measured in the VIGS plants. RNAs were extracted from three WT plants and from three plants for each VIGS type (*n*=3). Ubiquitin (Niben101Scf01956g01003.1) was used as internal control. **(A)** PDS silencing is effective in the co-silenced plants. The average PDS expression levels were calculated for WT and the VIGS plants, then the mean value of the WT plant was taken as 1 and the PDS mRNA expression levels of the different VIGS plants are shown relative to it. Note that the PDS silencing was comparably effective in the PDS-silenced as well as in the co-silenced plants. **(B-D)** VIGS selectively and efficiently reduced the levels of the target mRNAs. The average expression levels were calculated, then the mean value of the PDS-silenced sample was taken as 1 and the expression levels of the co-silenced plants are shown relative to it. Note that both the RST1 and RIPR silencing were selective and efficient although the SKI2 silencing was obviously more efficient. The results of the reporter assays strongly support that both the RST1 and RIPR silencing result in loss of function phenotype.

Supplementary figure 2: Alignment of the *Arabidopsi*s and *N. benthamiana* RST1 and RIPR proteins. **(A)** The predicted *N. benthamiana* RST1 protein was aligned to the Arabidopsis RST1. The annotated *Arabidopsi*s DUF3730 domains (82-352 and 537-748 amino acids) are yellow marked. **(B)** The predicted *N. benthamiana* RIPR1 protein was aligned to the *Arabidopsis* RIPR1. The two disordered regions of *Arabidopsi*s RIPR protein (1-60 and 296-315 amino acids) are blue marked. Needle pairwise alignment tool was used for sequence comparisons (https://www.ebi.ac.uk/Tools/psa/emboss_needle/).

Supplementary figure 3: NGD is induced in a position-dependent manner in plants. **(A)** Non-proportional representation of P281 and P95 NGD reporter transcripts. In the P281 NGD reporter mRNA a 36A stretch separates the 281 nt long PHA segment from the GFP. The 5' UTR was extended by a stuffer segment. P95 is a deletion derivative of P281, the PHA segment was shortened to 95 nt. P197 and P146 constructs (not shown) are identical except

the length of the PHA segment. Note that reporter transcripts are shown. **(B)** NGD efficiency depends on the position of the NGD inducing A-stretch. Reporter genes were co-agroinfiltrated with P14 (left panel) or with P14 and Pelota2 (Pel2), a dominant-negative version of Pelota (right panel). Three plants were agroinfiltrated with each mixture (*n*=3) and RNA was isolated from one agroinfiltrated leaf of each plant. RNA gel blots were hybridized with stuffer 5' UTR probes (upper panel). Ethidium-bromide (EtBr) stained gels are shown as loading control (bottom panel). Full-L. shows the full-length mRNAs, while 5' cleav. indicates the NGD generated and Pel2 stabilized 5'cleavage fragments. An RNA gel blot showing one set of samples is presented at Fig. 1B. Quantification is described and the result of the quantification is shown at Fig. 1B.

Supplementary figure 4: RST1 and RIPR proteins are involved in plant NGD and NSD. **(A)** Non-proportional representation of the PHA-72A-GFP (P-72A-G) NGD and the PHAnonstop (PHAnst) NSD reporter transcripts. **(B)** The RST1 and the RIPR are required for the decay of the 5' fragments generated by NGD. P-72A-G NGD reporter construct was co-agroinfiltrated with P14 into a leaf of three PDS, PDS + SKI2 (P+SKI2), PDS + RST1 (P+RST1) and PDS + RIPR (P+RIPR) VIGS plants (*n*=3). RNA gel blots were hybridized with P14 and PHA probes (probes are in italics). P-72A-G indicates the full-length reporter mRNAs, while 5' cleav. marks the 5' NGD cleavage fragments. An RNA gel blot showing one set of samples is presented at Fig. 2C. Quantification is described and the result of the quantification is shown at Fig. 2C. **(C)** The RST1 and the RIPR are required for the elimination of NSD target transcript. The PHAnonstop (PHAnst) NSD reporter construct was co-agroinfiltrated with P14 into three PDS, P+SKI2, P+RST1 and P+RIPR VIGS plants. PHAnst indicates the nonstop reporter mRNAs, while P14 shows the silencing suppressor transcript. An RNA gel blot showing one set of samples is presented at Fig. 2D. Quantification is described and the result of the quantification is shown at Fig. 2D. Ethidium-bromide (EtBr) stained gels are shown as loading control (bottom panel).

Supplementary figure 5: RST1 and RIPR proteins play a role in the elimination of 5' cleavage fragment of miRNA- and viral siRNA-programmed RISC. **(A)** Non-proportional representation of the reporter transcripts. **(B-C)** The RST1 and the RIPR are involved in the degradation of the 5' fragments of miRISC or vsiRISC. Three PDS, PDS + SKI2 (P+SKI2), PDS + RST1 (P+RST1) and PDS + RIPR (P+RIPR) VIGS plants (*n*=3) were co-agroinfiltrated with GFP miRISC reporter, with amiRGFP and P14 **(B)**, or with PHA-PDS-GFP (PPG) vsiRISC reporter and P14 **(C)**. GFP and PPG indicate the full-length reporter mRNAs, while 5' cleav. shows the 5' cleavage fragments. RNA gel blots were quantified as described at Fig. 2C and the results of the quantifications are shown at Fig. 3. RNA gel blots showing one set of samples are presented at Fig. 3. Ethidium-bromide (EtBr) stained gels are shown as loading control (bottom panel).

Supplementary figure 6: The conserved uORFs did not induce endonucleolytic cleavage in agroinfiltration assay. **(A)** Non-proportional representation of the 5'UTR testing system. 5'UTR region of the four conserved uORF containing genes and 5'UTR of the NIP5 Minimum ORF containing gene were cloned upstream of the GFP. The 5'UTR were extended by incorporating a stuffer segment (5' stuffer) upstream from the tested 5'UTRs. Red box indicates the conserved uORF. **(B)** The PHA-36A-GFP (P-36A-G) control NGD reporter construct, the four conserved uORF (780, HsfB, S30 and HB) and the Minimum uORF (MuO-G) containing reporter constructs were co-agroinfiltrated with P14 into PDS and PDS+XRN4 (P+XRN4) VIGS plants. P14 and GFP probes (italics) were used for the gel blots. The P14 transcript, the different full-length GFP containing reporter mRNAs (Full-L.)

and their 3’ cleavage (3’ cleav.) fragments are marked. Note that 3’ cleavage fragments accumulated to easily detectable levels only in the P+XRN4 VIGS plants (right panel) that were agroinfiltrated with the P-36A-G NGD positive control and the MuO-G reporter constructs. Ethidium-bromide (EtBr) stained gels are shown as loading control (bottom panel).

Supplementary figure 7: The AUG of Minimum ORF is essential for the cleavage. **(A)** Non-proportional representation of the reporter transcripts used in this experiment. MuO-G is the Minimum uORF reporter transcript. No-uO-G is identical except that the start codon of the Minimum ORF was eliminated. **(B)** The MuO-G and No-uO-G reporters were co-agroinfiltrated with P14 into *N. benthamiana* leaves, and then the GFP activity was assessed by UV illuminating the leaves. Note that elimination of Minimum ORF dramatically enhanced the expression of the GFP major gene. **(C)** The MuO-G (left panel) and No-uO-G (right panel) reporters were co-agroinfiltrated with P14 into a leaf of three PDS, PDS+XRN4 (P+XRN4), PDS+SKI2 (P+SKI2) and PDS+Pelota (P+Pel) VIGS plants. Stuffer (upper panels) probes were used to visualize the full-length mRNAs (MuO-G and No-uO-G) and their 5’ cleavage (5’ cleav.) fragments, while GFP (middle panels) probes were used to visualize the MuO-G and No-uO-G full-length mRNAs and their 3’ cleavage (5’ and 3’ cleav.) fragments. Note that the 5’ cleavage fragments of Mu-O-G samples (C, left upper panel) are detectable in all plants but they are dramatically overaccumulated in the P+SKI2 VIGS plants. The left panels are also shown as main figure (Fig. 4B), here are presented only for comparison. Ethidium-bromide (EtBr) stained gels are shown as loading control (bottom panel).

Supplementary figure 8: Inactivation of Pelota and HBS1 do not lead to the accumulation of the Minimum ORF induced 5’ cleavage fragments. **(A)** Non-proportional representation of the reporter transcripts used in this experiment. **(B)** The P281 NGD and the MuO-G minimum ORF reporter constructs were co-agroinfiltrated with only P14 (-) or with P14 and the *Arabidopsis* Pelota (Pel1) or with P14 and the dominant-negative paralog of Pelota (Pel2). Pel2 inhibits the Pelota-HBS1 complex. Full-L. indicates the full-length P281 and MuO-G transcripts, while 5’cleav. shows the 5’ cleavage fragments. Red star and exclamation mark indicate the 5’ cleavage fragment of P281 and MuO-G, respectively. Note that Pel2 co-infiltration did not lead to the overaccumulation of 5’cleavage product (5’cleav.) of MuO-G minimum ORF reporter transcript (relative to the – and Pel1 samples) but led to the dramatic overaccumulation of the 5’ cleavage fragment of the P281 NGD reporter mRNA. **(C)** Silencing of HBS1 does not result in the accumulation of the 5’ cleavage product of MuO-G minimum ORF reporter transcript. The P281 NGD and the MuO-G minimum ORF reporter constructs were co-agroinfiltrated with P14 into a leaf of two PDS and PDS+HBS (P+HBS1) VIGS plants. Note that the 5’cleavage fragments of P281 NGD accumulate to strongly enhanced levels in P+HBS1 plants relative to the PDS control lines (compare lanes 5-6 to 1-2), while the 5’ cleavage products of MuO-G accumulate to comparable levels in PDS and P+HBS1 VIGS plants (compare lanes 7-8 to 3-4). Note that only 2-2 samples were studied.

Supplementary figure 9: The role of RST1 and RIPR in the degradation of 5’ cleavage fragments of Minimum ORF. **(A)** Non-proportional representation of the Minimum ORF reporter mRNA (MuO-G). **(B)** The RST1 and the RIPR are required for the elimination of the 5’ fragments of Minimum ORF induced cleavage. MuO-G was co-agroinfiltrated with P14 into PDS, P+SKI2, PDS + RST1 (P+RST1) and PDS + RIPR (P+RIPR) VIGS plants (*n*=3). The RNA gel blots were quantified as described at Fig. 2C. The results of the quantifications are shown at Fig. 4C. RNA gel blots showing one set of samples are presented at Fig. 4C.

Ethidium-bromide (EtBr) stained gels are shown as loading control (bottom panel). Note that the 5'cleavage fragment of MuO-G accumulates to low but detectable levels even in PDS VIGS plants.

Supplementary figure 1: The target mRNA levels are efficiently reduced in the VIGS plants.

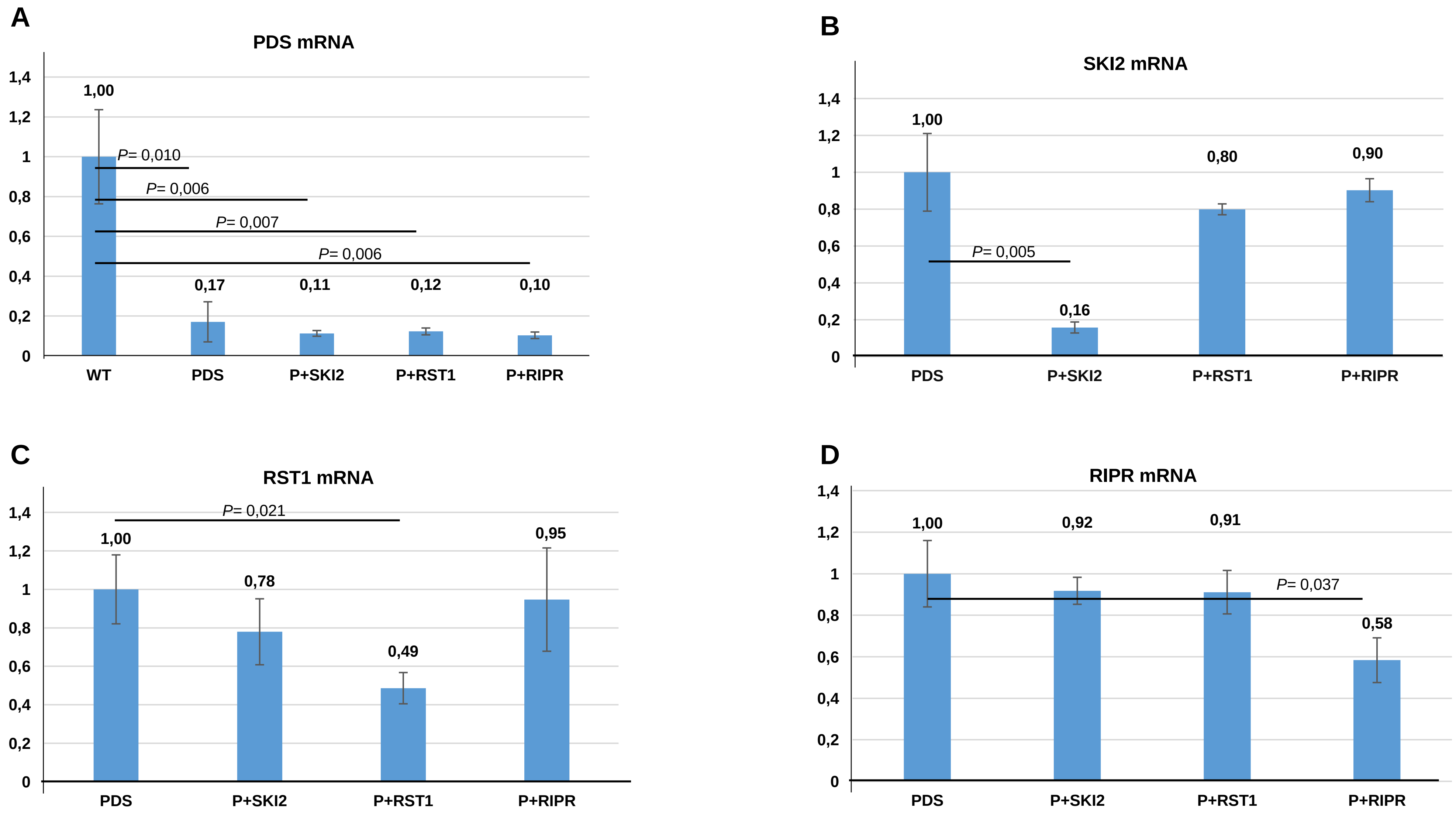

Supplementary figure 2: Alignment of the *Arabidopsis* and *N. benthamiana* RST1 and RIPR proteins.

Aligned_sequences: AT RST1:NB RST1# Length:1912# Identity:45.1%# Similarity:62.4%

A

```
AT RST1      1 MASYATLLEKTRVPQPSIQRFAVISVFSKLRSAPEQFGSEAEAGREAISF     50
               |.||..||||.|:||||:|:|||||:|.||||||....|::..|.:||:.
NB RST1      1 MDSYTQLLEKIRIPQPSLQKFAVISIFEKLRSAPPHLDSDSAPGTDAITQ     50

AT RST1     51 CLTSESITVVDQSVHELCRLVSDSVLDLSRGLLELQSALEGCDSKLVSLF    100
               ||.|.|.:|:||||.||||||.||.||:||||||||||||||.||:.|:||
NB RST1     51 CLHSTSASVLDQSVRELCRLVRDSKLDISRGLLELQSALEGSDSRFVNLF    100

AT RST1    101 VKGLGFLIRIGYERNNGNWKFNSTENHPFVRIQSSRVETQTELLHQVSLF    150
               |||:|||:|:|:::|  :.:|.::|.||||::.|.|||.||||:.||.:|
NB RST1    101 VKGIGFLVRLGFQKN--SLRFLTSETHPFVKVLSCRVEVQTELVQQVIIF    148

AT RST1    151 VMHNRRLGMVGVCEFLEPFLNFTILRIPLSDSSSSLFARELISSMASLCC    200
               ::..:.||||..||||.|.||::|:|:|.|.|.|| |.:.||||:|.|||
NB RST1    149 IVQRKHLGMVEACEFLVPVLNYSIVRMPSSVSLSS-FIKSLISSLAGLCC    197

AT RST1    201 SSRHEALPIFRLLMRCLKYIPGNNLEVIVKI------LVDAYTVVVRDLV    244
               |...||:.|..||:..||:.|.||.|....|      :||||.||::.||
NB RST1    198 SIPGEAISIIELLIGRLKFFPCNNSEDFTNISHCLECIVDAYVVVLQQLV    247

AT RST1    245 GTGLEVTEVHLLGVQLVDGVLFLCASPHVQTTEQESVIESLKHLLAVQKD    294
               ..|..:.|..|.||.|:|.:  ||.:|. .|:..|:::|..|.:|.||||
NB RST1    248 EVGSLLHEAQLCGVGLLDAM--LCMNPK-HTSSVENILEVSKRILVVQKD    294

AT RST1    295 LGLAYSHDLSLVVLSLVFMLAKSTVEHEQLCILKFLLFLLKWKTESENLS    344
               ||..|...||.:.|||..:|.:|.:||||..::|.:|||||||.|:|:..
NB RST1    295 LGFGYLPQLSTITLSLFMILVQSELEHEQFLVVKLILFLLKWKYENEHDV    344

AT RST1    345 VKDAAGSSVESLLLFPITALMSSPSKSIKVAASKVLSI---VENFLVTVS    391
               .:||.....|.|.:||..:|:|||||.:|.||:.:|.|   :.|.|:|..
NB RST1    345 QRDACDLKEELLFIFPAISLLSSPSKIVKQAATDLLHILGKLSNKLLTAQ    394

AT RST1    392 -----NAPKI-EVHTSKGDSPLSRVGSVVFRFMQQLWHQNDYTPSTSSFL    435
                    ||.|. .:.|.|         .:|||.:|.||.| |.:|.:.||
NB RST1    395 KTGQPNAMKFPSISTPK---------YIVFRLLQHLWLQ-DLSPLSGSF-    433

AT RST1    436 RVAYTN-----GSEKQETYLGPVTWNSLLREHAERFWDKKKLSASFCLSQ    480
                  |.|     .:..::|:....||:||:.:|......::| |.|...||
NB RST1    434 ---YPNYVPGHDTSIKDTHYVSKTWSSLVTDHMHHIIARRK-SLSISQSQ    479

AT RST1    481 EI-----PILLGAVAGVMVMHPSLGADAIGSLTIIGGIDSKMSVPLLLAV    525
               ||     |::..|||.|::.|.:.|:.::..|:....:|.|:.||||||.:
NB RST1    480 EIFPTNMPMIFSAVACVLLTHQTYGSSSVDILSNCSNVDPKLGVPLLLVI    529

AT RST1    526 LYFSNLLS-RTNVPCQSLLSKLLGLLPSLAAQQVMIPLVVQTITPMLRKD    574
               .:::::.| .|...|..:|.|||.:||.||:...:|||::||:.|||:.|
NB RST1    530 QFYNHIFSTNTGADCHGVLLKLLEMLPLLASHPAIIPLIIQTLLPMLQND    579

AT RST1    575 AKGLLYATAIRLLCQTWVVNDRAFSSLQEVLRPQGFIEYISERHICISMA    624
               .|.:|:|||||||||:||.:|||
NB RST1    580 KKPVLFATAIRLLCKTWELNDR----------------------------    601

AT RST1    625 ASIHDVCKRHPDRGVDLILSVQACIESQNCPVRALGFQSLSHLCEADVID    674
                             :||                 ||.    ||.|...:.
NB RST1    602 --------------IDL-----------------LGL----HLREISALV    616

AT RST1    675 FYTAWDVIKKHAQHIKLDPLLAYSVCHLLKWGAMDAEAYPEDAENVLNIL    724
               :...:        .|..:.:|...:|.||.||||||:||||.:.|||.||
NB RST1    617 WLFPF--------VISAEEILIVDLCLLLTWGAMDAQAYPEASVNVLKIL    658

AT RST1    725 WEIGSSMQKPHDSQWTKARVSAIVALGQYEVSFMENKFSDF-NKNCTYLL    773
               |:||:|......|.|:|||.||.|||..|||..:|....|| :||..:|:
NB RST1    659 WDIGTSQDFRQASLWSKARASAFVALASYEVEHLERSIPDFKDKNLEFLV    708

AT RST1    774 FSETNAEILNALEDLSIKIMIHEHSVRRRYVREKKVPGSKIEKLLDVIPQ    823
                |||:.|:|.|:|...:||:..||:.|||.|::|:|..:||||||||.|:
NB RST1    709 -SETDPEVLTAVEGFEVKILTFEHTTRRRLVKQKRVSANKIEKLLDVFPR    757

AT RST1    824 VIFPAGKEIKTGELPGAALLCLSYNPRDV-KFGSSRSFHDVHFQYEEAFR    872
               :||.:|||.:..|||||||.|||:..:|. |.|::....||..:|:.:..
NB RST1    758 LIFASGKERREKELPGAALFCLSFTKKDSRKAGAAEDLQDVQAKYKASLV    807
```

Supplementary figure 2: Alignment of the *Arabidopsis* and *N. benthamiana* RST1 and RIPR proteins.

A

Aligned_sequences: AT RST1:NB RST1# Length:1912# Identity:45.1%# Similarity:62.4%

```
AT RST1          873 VVVKSLQLSRNISLALISLQSLKAFMRRWMRANILSIDATTKELSSDKTS    922
                     .:..||||||||.:|::||||.|.|||||||:||.:||..:....||..
NB RST1          808 DIATSLQLSRNILIAILSLQSWKPFMRRWMRAHILLLDAKLQTAVLDKAP    857

AT RST1          923 KATNNIMKSLVHMAEEALPRCAENIALALGALCAALPAASHNIKASASKF    972
                     ||...|:|:::.:||..|||.||||||||:||||..||||::|.:||:||||
NB RST1          858 KAGMEILKTMIAIAERMLPRSAENIALAVGALCLVLPASAHVVKATASKF    907

AT RST1          973 LLSWLLEHEHEHRQWTAGISLGLISSSLHVTDHKQKFQNISGLLEVLCSS   1022
                     ||.||.:||||:||.:|.||||:|||.||:||||||||:||:.||||...|
NB RST1          908 LLDWLSQHEHEYRQCSAAISLGVISSCLHLTDHKQKFENINALLEVASVS    957

AT RST1         1023 KSTLVKGACGVGLGFSCQDLLTRTEASASSDIDSDSYRNQEERLLGRIVR   1072
                     |:||||||||.|||||||.||.|.:|.::.:.:....|:.:|..||.:|:|
NB RST1          958 KNTLVKGACGAGLGFSCQALLARADADDNAHLGKARYKIEEADLLRKIIR   1007

AT RST1         1073 LLSSILHGFLHTPCDILESLSALFPPGEEDNV-------IGLPQLLDESS   1115
                     .||.::.....:..|:.|:||..||. |.||:       :||      :|
NB RST1         1008 TLSQLICQVSPSSADVFETLSLSFPL-ESDNLNSEISGSLGL------TS   1050

AT RST1         1116 DDFDDDTWGIAGLIIGLGMSVGAIYRAGKKDAVVKIKNLIVSWIPYADSL   1165
                     ::.::|.||||||::|||..:||:||||..:||:.:|:|::||||:...:
NB RST1         1051 ENLEEDVWGIAGLVLGLGNCIGAMYRAGIHNAVLNVKSLLISWIPHPTEI   1100

AT RST1         1166 KQTSGSNSKVSVRLFSVGSCLALPIVITFCQKVELFDAHEVDDIIGCFKD   1215
                     ...| .|.::   |.||||||::||||..||:.||.|..:::.::||:|:
NB RST1         1101 TAMS-KNHEI---LLSVGSCLSVPIVIAMCQRFELTDDADMEYLLGCYKE   1146

AT RST1         1216 LISELLIVRKSGALRKRLLMASCIGAGDLLGSVLNEGIHPVKIESVKELL   1265
                     :|||||.|::.....:.||||||:|||.|:|.||||||:||:|||.:||||
NB RST1         1147 IISELLSVKRFDTFHQSLLMASCLGAGSLIGVVLNEGLHPLKIEHIKELL   1196

AT RST1         1266 ELFKKCYSGLYPPVAHFGGMLGVVNVLGAGAGNLVYSHPRPRAPPASSEE   1315
                     .||:|.||...||:.|.|.|||:||.||||||.||..||. .:..|:|:.
NB RST1         1197 LLFRKSYSDSNPPLIHLGAMLGIVNALGAGAGTLVEPHPL-SSSHAASDL   1245

AT RST1         1316 NEISYVSGPLLSNAYFTQQLTPVVQEIFLIAQNTKDRQLQHYAAWAISIL   1365
                     .|.||:|.||::||....:||.:||||||:|||:...|||.|||||||.|
NB RST1         1246 KESSYISSPLITNAVLEPELTSLVQEIFLVAQNSDAHQLQQYAAWAISFL   1295

AT RST1         1366 RTYMRSGESSSLSNENQSDDSDRNSISHNVPEHTMVMKLAQGLTNPSFPL   1415
                     |.::...|..:.....::|.:...::.|:.||.:.||||:..|.:.::..
NB RST1         1296 RQHLWFKELQNDETTAENDSAGLKTVLHSFPEDSTVMKLSLWLMHLNYLG   1345

AT RST1         1416 AGSPLNIGTMASALRCLSHAPRLPNLDWGATIRRLMKQETQTDVTQSGDV   1465
                     ..:..::.|::|.|||||||.|||.||||||.|||.|:.|.....:.|:
NB RST1         1346 TDAVSHVNTVSSVLRCLSHASRLPPLDWGAIIRRCMRYEGQVAGLLTQDI   1395

AT RST1         1466 PKEI-TLREECFKFSLAHASEFDELLAFLDELSELSRFKALEESLQSCLL   1514
                     ..|. .||.||..|||:||.:||.||:||||..::.|.:.||..||..||
NB RST1         1396 SFERGNLRGECILFSLSHAKQFDPLLSFLDEQCDIPRLRMLEPRLQFFLL   1445

AT RST1         1515 CHLGGLMRIFSGSRMNKLFDDVSCFV-VSLSSDQTYSCD--QKSSLRVSC   1561
                     .||..|::||||||:.|||:||:..: .|:.|:   |||  :|||.|:||
NB RST1         1446 SHLADLVKIFSGSRVVKLFEDVANLLSTSICSE---SCDSLEKSSFRISC   1492

AT RST1         1562 WKGLSQCLEETSLESSEYVTKIEKCIELLFAVLPVASQSPRADQMGSVKE   1611
                     |:||..||:|:.::..||.:.|||:||||..|| ::.:....|...::|
NB RST1         1493 WRGLKLCLDESSHHTQEYKSSIEKCMELLFTSLP-SAHTEGLCQGKILEE   1541

AT RST1         1612 WSEAVTCLQKSHRDWLYKFLQVSNLEPGNEKTNFQGDLKKIQAKAKLAKL   1661
                     |.||:.||:|:.::||...|:||:.:...:.:....::||||||||.:.
NB RST1         1542 WCEAIRCLEKAQQEWLLDLLKVSEVNVADSDSLSFETVRKIQAKAKLVQS   1591

AT RST1         1662 GSVPFSELGKLKAIILNCEESDIWDVLIEIVAALHHAEGGIKRQWLIDAV   1711
                     ||:|.:.|||||..:|:|..:||||.||||:...:.||||..|||||||:|:
NB RST1         1592 GSLPLTVLGKLKTYLLDCRSQDIWDALIEVSITVQHAEGNAKRQWLIEAL   1641

AT RST1         1712 EISCVSSHPSTAIIFVGLLSSICCEYMPFLNLDRSTVLSDMSVTVTSLLS   1761
                     |||||:..||||:.|:|||...||.|.|.|..|:.||||||:.||:|||||
NB RST1         1642 EISCVTRFPSTALQFIGLLCGSCCVYRPVLFADKFTVLSDLPVTLTSLLS   1691

AT RST1         1762 DPSYEVVTEPFISFLWTSLERVYSF--ATESDANARLSSQQIAQSERDKA   1809
                     |.::.||.:..:|.||.|.||:|.:  ..:..::..|.:|.|.|..||.|.|
NB RST1         1692 DSTWMVVADAVVSCLWKSTERIYKWNEQLKGGSDYLLYTQPIDTSENDIA   1741

AT RST1         1810 PMLVKVMHYICVAFRDHLPLEKQLRLASMDMS*-----------------   1842
                     ..|:.|||..||:.:||||.||||:||:.:.:..
NB RST1         1742 CFLLLVMHQACVSLKDHLPPEKQLQLANMVVPSNVDAHELHARWKTIFMD   1791

AT RST1         1843 ------------   1842

NB RST1         1792 VCEQLSDDVMN*   1803
```

Supplementary figure 2: Alignment of the *Arabidopsis* and *N. benthamiana* RST1 and RIPR proteins.

B

Aligned_sequences:AT RIPR:NB RIPR# Length:447# Identity:32.2%# Similarity:48.1%

```
AT RIPR            1 MDSKSLAKSKRAHTLHHSKKSHSVHKPKV----PGVSEKNPEKLQGNQTK     46
                     ||:|:||||||||:||.:||.:..|..|.    .|.|....:|..|.|.|
NB RIPR            1 MDAKALAKSKRAHSLHLNKKHNPHHAFKASSAGSGASTTGDKKPTGKQVK     50

AT RIPR           47 SPVQSRRVSALPSNWDRYDDELDAAEDS------SISLHSDVIVPKSKGA     90
                     ...:|:    ||||||||::|.  |.||      ..:..|||:||:||||
NB RIPR           51 EKPKSK----LPSNWDRYEEEY--AFDSENVPQGGANKASDVVVPRSKGA     94

AT RIPR           91 DYLHLISEAQAESNSKIENNLDCLSSLDDLLHDEFSRVVGSMISARGEGI    140
                     ||.:|:|||||:    .:::.:.:...||.|        |.::||:|:..
NB RIPR           95 DYAYLLSEAQAQ----FQHSSESIPLYDDGL--------GELLSAKGQSK    132

AT RIPR          141 LSWMEDDNFVVEEDGSGSYQEPGFLSLNLNVLAKTLENVDLHERLYIDPD    190
                     |||:.||||.: ||.:....:..||||:|:.|::.||...|.|||:|:||
NB RIPR          133 LSWIADDNFAM-EDKAPPPTKASFLSLDLHALSEQLERASLSERLFIEPD    181

AT RIPR          191 LLPL-PELNTSQ----------------------------TKV-----SR    206
                     |||| |.:..||                            |.|     :|
NB RIPR          182 LLPLEPCIEASQAAAEEKRQGGLSSSKSSTAEKYSNSLTSTSVYEGNKNR    231

AT RIPR          207 NEEPSHSHIA--------------QNDPIVVPGESSVREAESLDQVKDIL    242
                     ::....||:.              .::|:...|:.:.:...:  .:.|.|
NB RIPR          232 HQHSEFSHLGTTTSSSWHPTSADEPSNPLSAYGDEAGKSEGA--GINDSL    279

AT RIPR          243 ILTDESEKSSAI-----EADLDLLLNSFSE--------AHTQPNPVAS--    277
                     :  ..::||||.     ||:||:||:|.||        |..|.:|..|
NB RIPR          280 L--SVAKKSSAFKATVAEAELDMLLDSVSEIEIFESTNAIDQSSPSCSMA    327

AT RIPR          278 ASGKSSAFETELDSLLKSHSSTEQFNKPGNPSDQKIHMTGFNDVLDDLLE    327
                     .:|..:.......|.|:..|..::.:....|:   |.....:|.|||||.
NB RIPR          328 QAGTQTPLSEGTSSRLEDSSQPKRDHDLAKPA---ISDLSLDDSLDDLLR    374

AT RIPR          328 ST----------PVSI------IPQSNQ--TSSKVLDDFDSWLDTI*    356
                     .|          |:.:      .|.::|  :.||::||||||.||:
NB RIPR          375 ETSTVTNKNDGLPIQVNSTAGYTPSASQPVSKSKIMDDFDSWFDTL*    421
```

Supplementary figure 3: NGD is induced in a position-dependent manner in plants.

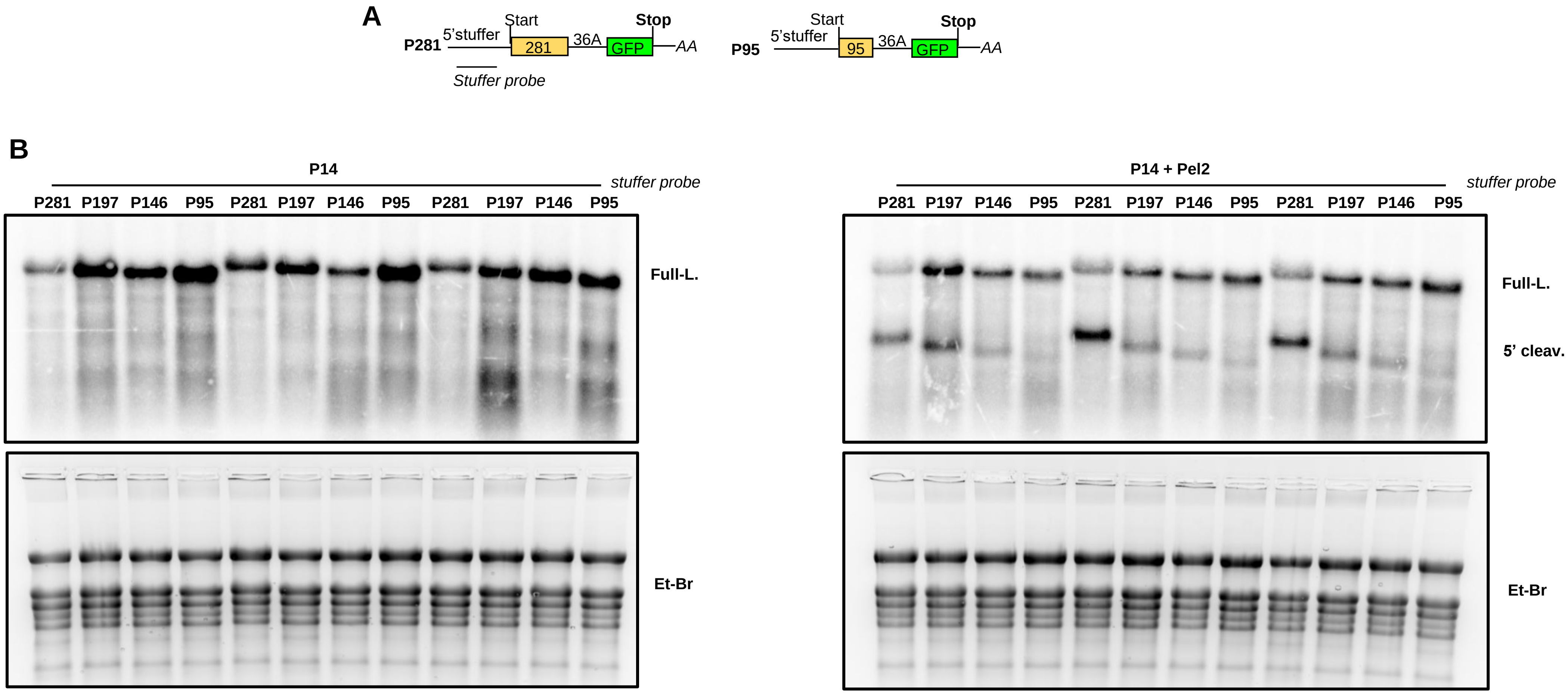

Supplementary figure 4: RST1 and RIPR proteins are involved in plant NGD and NSD.

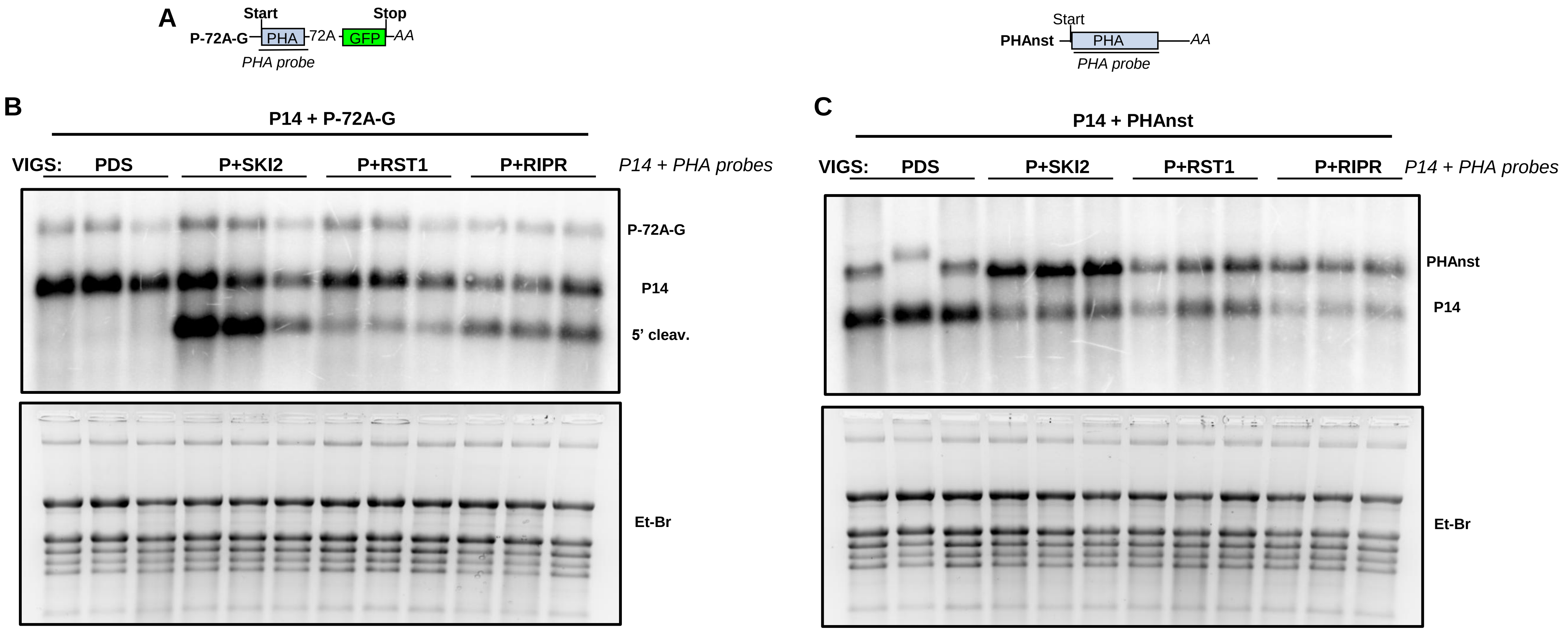

Supplementary figure 5: RST1 and RIPR proteins play a role in elimination of the 5’ cleavage fragment of miRNA- and viral siRNA-programmed RISC.

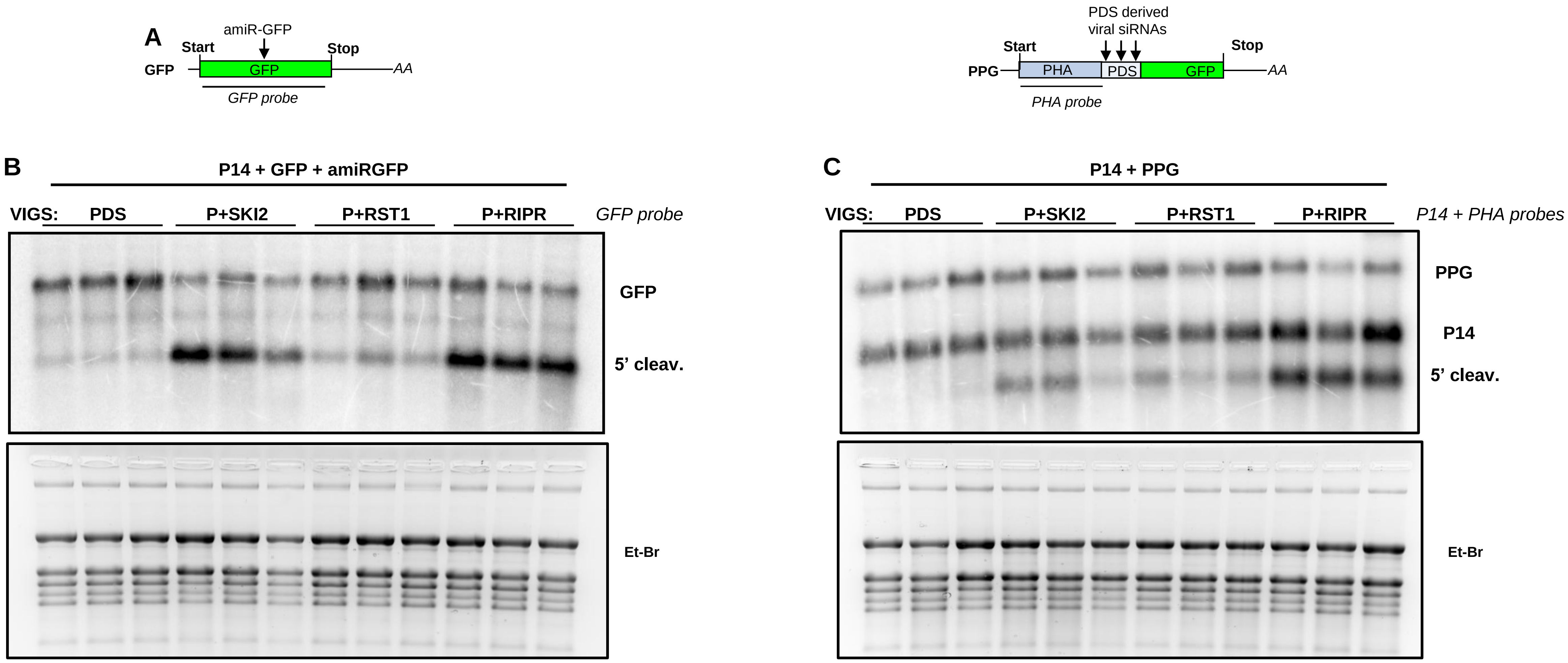

Supplementary figure 6: The conserved uORFs did not induce endonucleolytic cleavage in agroinfiltration assay.

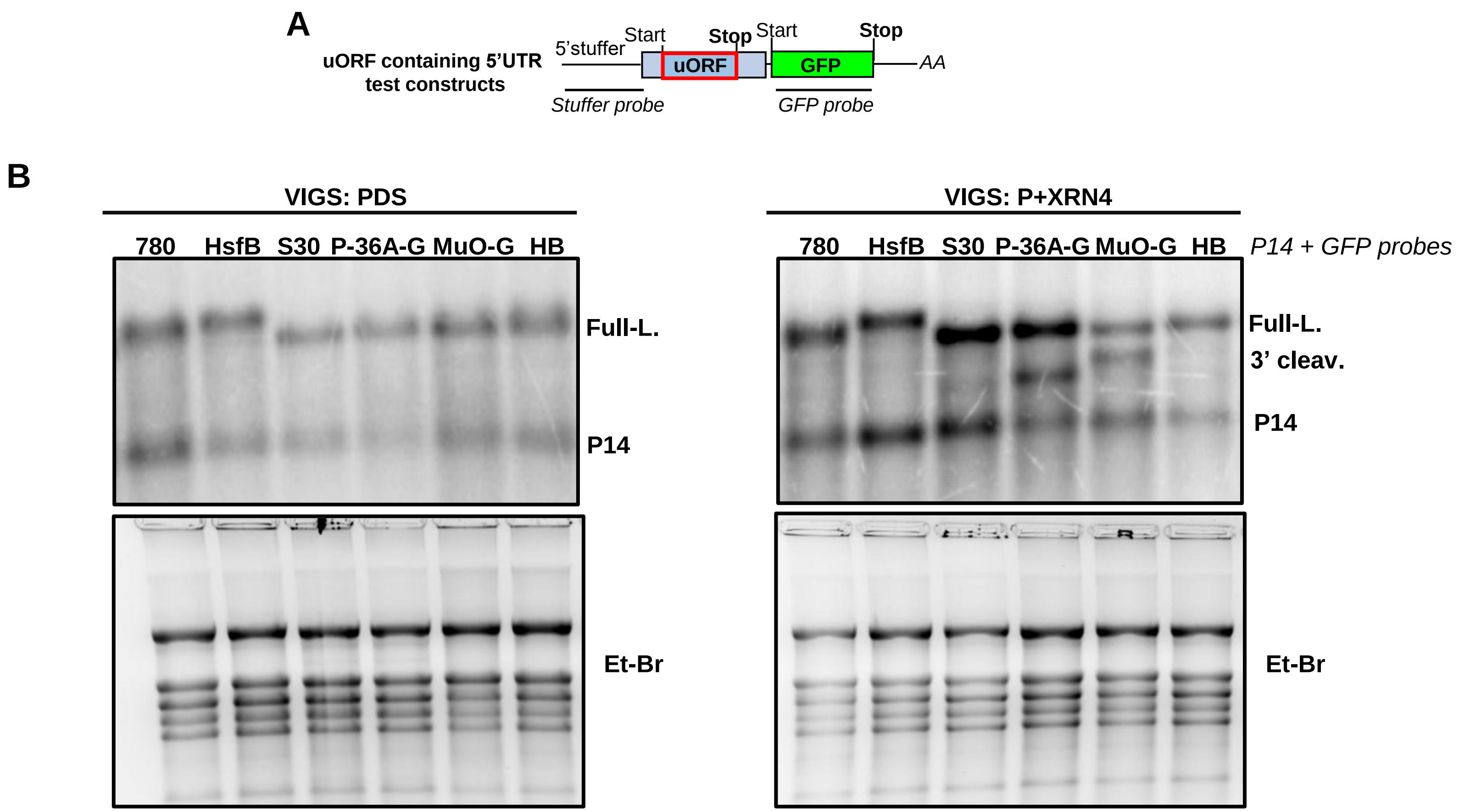

Supplementary figure 7: The AUG of Minimum ORF is essential for the cleavage.

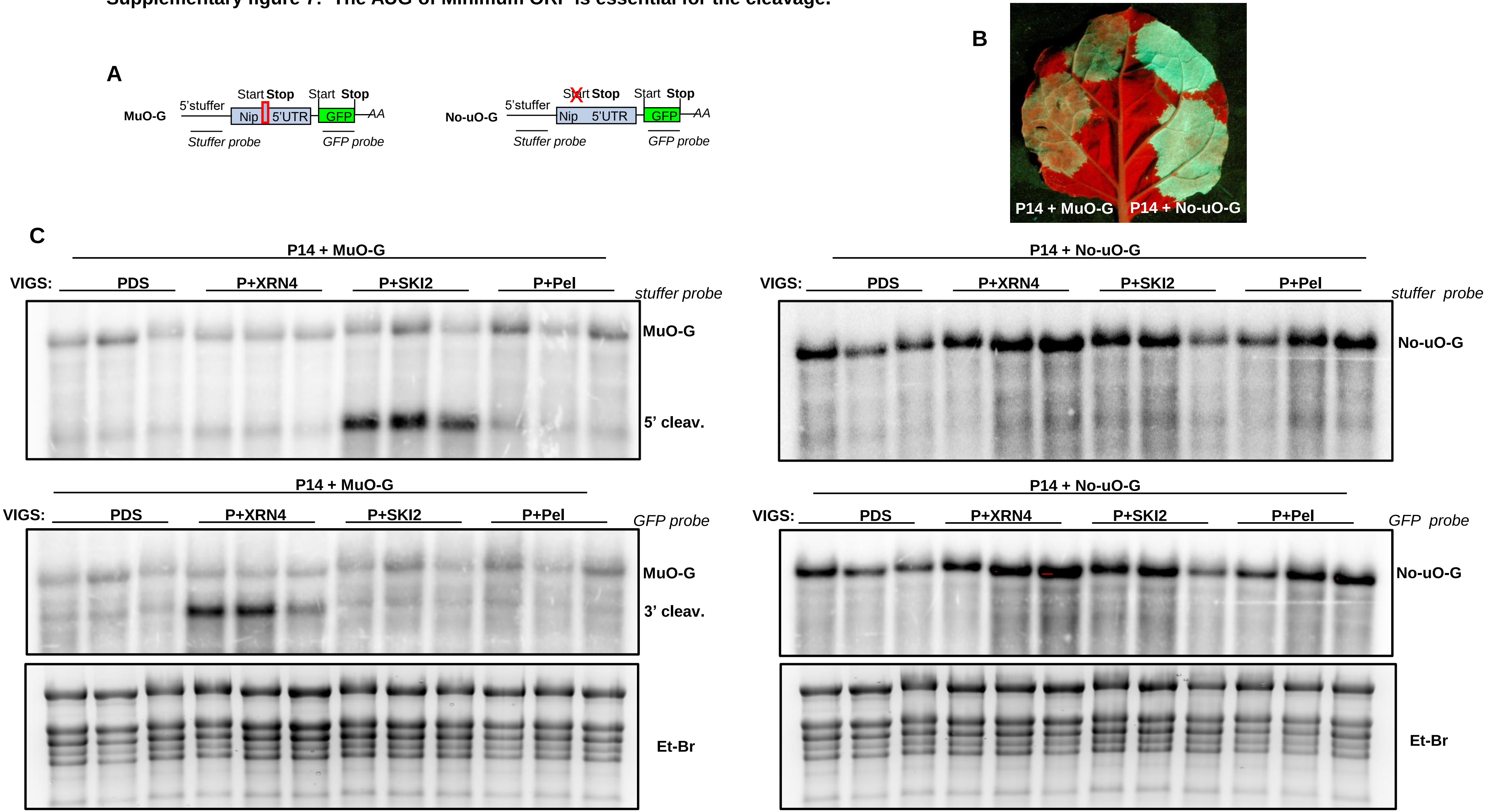

Supplementary figure 8: Inactivation of Pelota and HBS1 do not lead to the accumulation of the Minimum ORF induced 5' cleavage fragments.

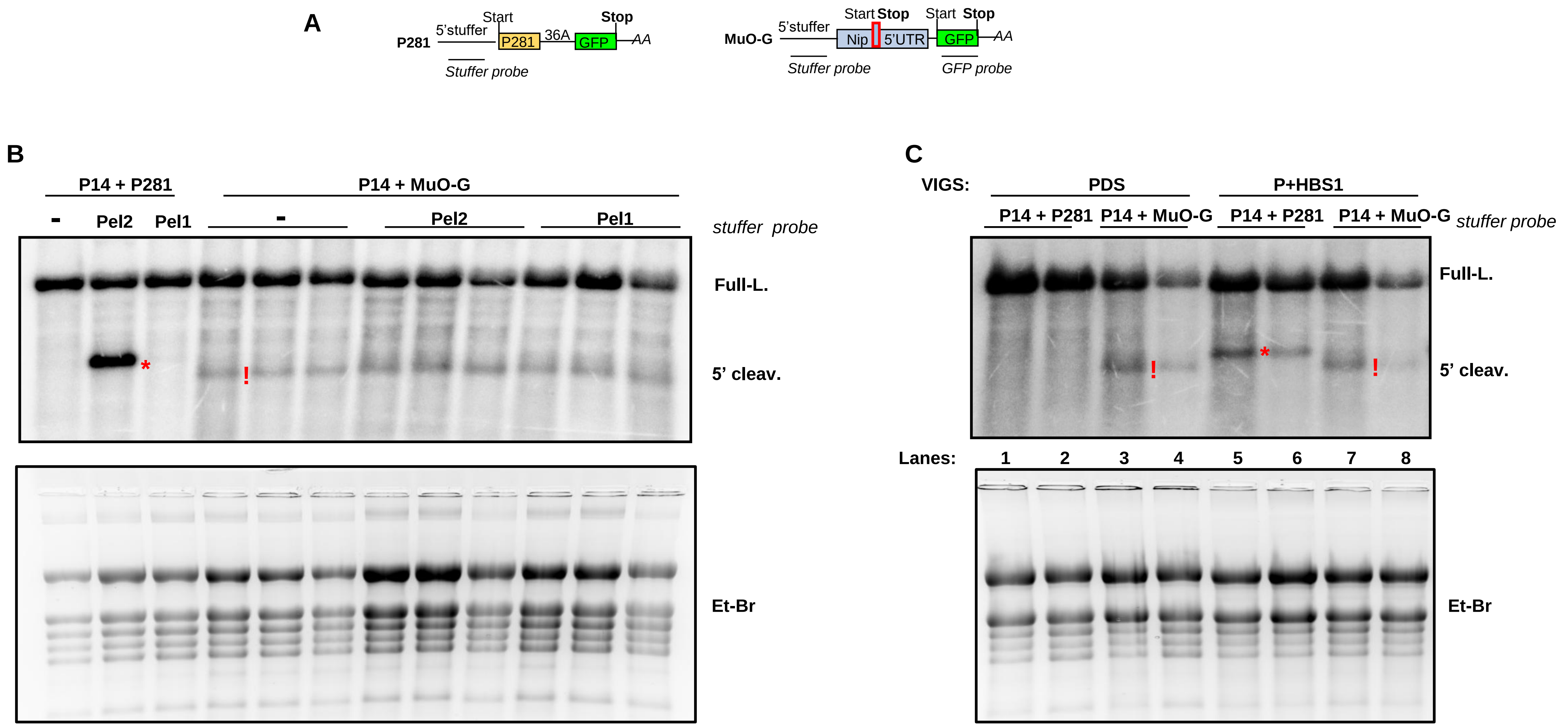

Supplementary figure 9: The role of RST1 and RIPR in the degradation of 5' cleavage fragments of Minimum ORF.

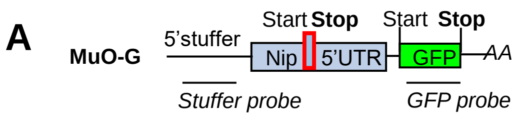

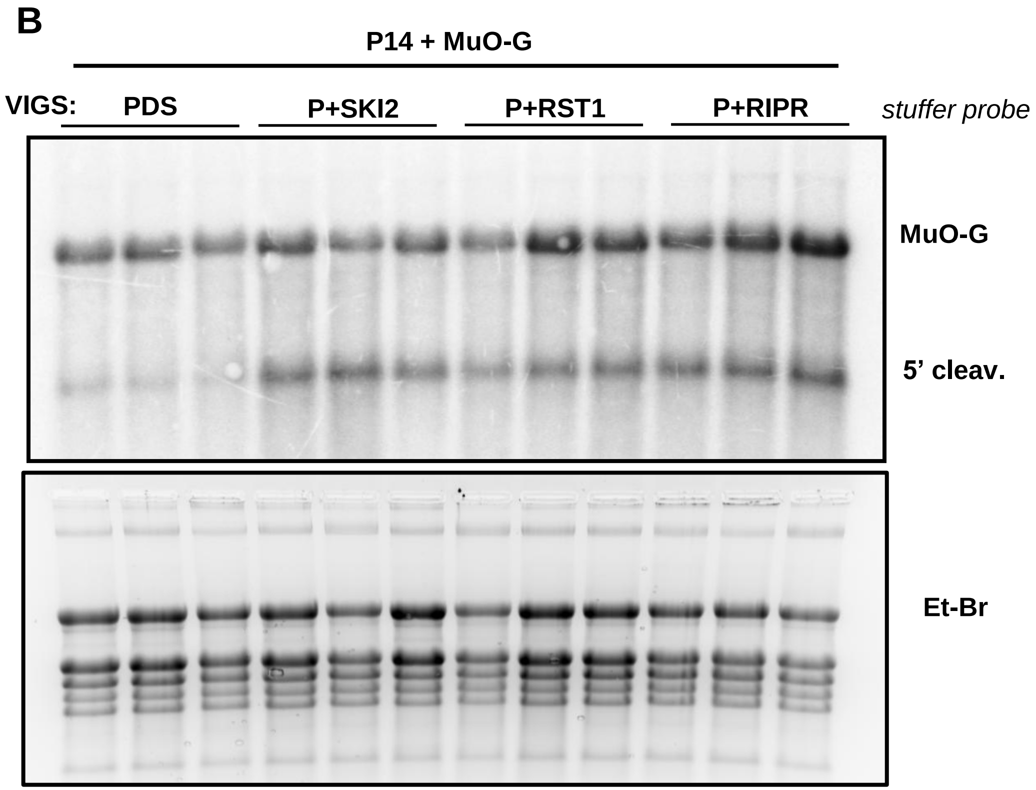

Supplement: Supplementary file 1 — Supplementary file1 (PDF 1155 KB) [file 11103_2021_1145_MOESM1_ESM.pdf]
